# Diagnostic Biomarkers and Metabolic Mechanisms of Polymyxin B-Induced Acute Kidney Injury

**DOI:** 10.3390/antibiotics15100966

**Published:** 2026-09-30

**Authors:** Sirui Zhao, Wei Qin, Zirui Zhao, Ying Liu, Wenqian Chen, Yue Zhang, Juan Ren, Liang Sun, Lizhi Chen, Jingjing Shen, Pengmei Li, Xiaoxue Wang

**Affiliations:** 1 Department of Pharmacy, China-Japan Friendship Hospital, No. 2 Yinghua Road, Beijing 100029, China; 2 Department of Pharmacy Administration and Clinical Pharmacy, School of Pharmaceutical Sciences, Peking University, Beijing 100191, China; 3 Institute of Chemicals Safety, Chinese Academy of Inspection and Quarantine, Beijing 100176, China; 4 Comprehensive Test Center, Chinese Academy of Inspection and Quarantine, Beijing 100123, China; 5 Department of Analysis and Testing, Shimadzu (China) Co., Ltd., Beijing 100020, China; 6 Beijing Key Laboratory of Critical Bridging Technologies for Chronic Disease Drug Development, China-Japan Friendship Hospital, Beijing 100029, China

**Keywords:** polymyxin B, acute kidney injury, biomarker, machine learning, therapeutic drug monitoring

## Abstract

Background/Objectives: The clinical utility of polymyxin B (PB), a critical last–line antibiotic for multidrug–resistant Gram–negative infections, is severely compromised by acute kidney injury (AKI). However, the lack of specific diagnostic biomarkers and the incomplete understanding of pathogenic mechanisms pose substantial challenges to clinical management of PB–associated AKI. Methods: We enrolled 83 patients receiving PB regimens (23 AKI, 60 non–AKI) and quantified the plasma concentrations of five PB components to identify the optimal therapeutic drug monitoring (TDM) metric for nephrotoxicity. A rat model of PB–induced AKI was established to map renal PB distribution via mass spectrometry imaging and screen targets for tubular accumulation virtually. Untargeted metabolomics was performed on clinical and rat samples to identify metabolic dysregulation, with key pathways validated by reverse transcription quantitative real–time PCR (RT–qPCR). Diagnostic models were constructed using the eXtreme Gradient Boosting (XGBoost) algorithm and evaluated using Area Under the Curve (AUC). Shapley Additive exPlanations (SHAP) analysis was applied to interpret the models, and decision curve analysis (DCA) was used to assess clinical utility. Results: A novel PB–component model outperformed the conventional TDM index (AUC 0.703 vs. 0.561), and a 6–metabolite exploratory classifier achieved 0.95 accuracy and AUC 1.000 for PB–AKI discrimination on an independent test set. Mechanistically, high PB concentrations might saturate P–glycoprotein (P–gp), leading to PB accumulation in renal tubules and subsequent metabolic dysregulation, manifested as upregulation of fatty acid pathways and downregulation of the TCA cycle. Conclusions: These findings advance our understanding of metabolic dysregulation in PB–induced AKI and support the potential utility of responsive metabolic features in the clinical diagnosis of PB-induced AKI.

## 1. Introduction

Multidrug resistance in Gram-negative bacteria is a growing challenge in the treatment of infectious diseases [1]. Although many antibacterial agents are no longer effective against multidrug-resistant pathogens, polymyxin B (PB) retains antibacterial activity, broad clinical availability, and favorable cost-effectiveness for drug-resistant infections [2]. However, the clinical utility of PB is severely compromised by dose-limiting nephrotoxicity [3]. The incidence of PB-induced acute kidney injury (AKI) can reach 30–60% and is closely associated with a marked increase in mortality risk [4]. Although conventional renal function tests and therapeutic drug monitoring (TDM) have been used as diagnostic and predictive tools for PB-induced AKI, the former are nonspecific and delayed [5], whereas the latter has limited accuracy [6]. PB exposure damages renal tubules and induces inflammation through mechanisms including mitochondrial dysfunction, oxidative stress, and activation of inflammatory cascades, ultimately leading to tubular cell death [3,7]. Current nephroprotective strategies, ranging from inhibition of renal PB accumulation to antioxidant therapy, have demonstrated limited clinical efficacy [3]. This persistent therapeutic gap suggests the existence of other mechanisms underlying PB-associated AKI that have yet to be elucidated.

Metabolic reprogramming has emerged as a hallmark of AKI, offering important opportunities for diagnostic, prognostic, and therapeutic applications [8]. Untargeted metabolomics enables the rapid and precise detection of differentially abundant metabolites in samples, facilitates systematic screening of the metabolome, and aids in identifying AKI-specific metabolites, elucidating disease mechanisms, and discovering therapeutic targets [9,10]. With the rapid development of artificial intelligence, particularly machine learning and deep learning algorithms, these methods have been widely used to develop predictive and prognostic models for various types of AKI [11]. Compared with traditional statistical methods, machine learning can integrate clinical, multi-omics, and laboratory data to improve diagnostic accuracy and efficiency, strengthen the evidence base for risk factor identification, and facilitate more precise treatment strategies [12]. However, such approaches have rarely been applied to PB-induced AKI. To date, only one urinary metabolomics analysis in rats has examined this condition and identified elevated taurine as a potential early biomarker [13]. Although that study advanced our understanding of metabolic alterations in PB-associated AKI, further metabolomics and machine learning investigations based on clinical cohorts are warranted to improve the diagnostic accuracy of PB-induced AKI and help elucidate its metabolic mechanisms.

TDM is recommended for critically ill patients to optimize PB dosing and reduce the risks of resistance and nephrotoxicity [14]. PB comprises five major active constituents: polymyxin B1 (PB1), B2 (PB2), B3 (PB3), isoleucine-polymyxin B1 (ILE-PB1), and B6 (PB6) [3], which have varying nephrotoxic potentials [15]. The International Consensus Guidelines on TDM identify the magnitude of PB exposure, represented by the area under the concentration-time curve within 24 h at steady state (AUC_ss,24h_), as the primary risk factor for PB-induced AKI [16,17]. However, the studies underlying the guidelines were based solely on total plasma PB concentrations and did not consider the concentrations of individual components. A clinical study demonstrated that the peak concentration (C_max_) of PB1 was a stronger independent predictor of nephrotoxicity than AUC_ss,24h_ [18]. Thus, whether AUC_ss,24h_ is the optimal TDM metric for predicting PB nephrotoxicity remains controversial, necessitating a reassessment of component-specific exposure thresholds to refine TDM strategies.

**Figure 1:**
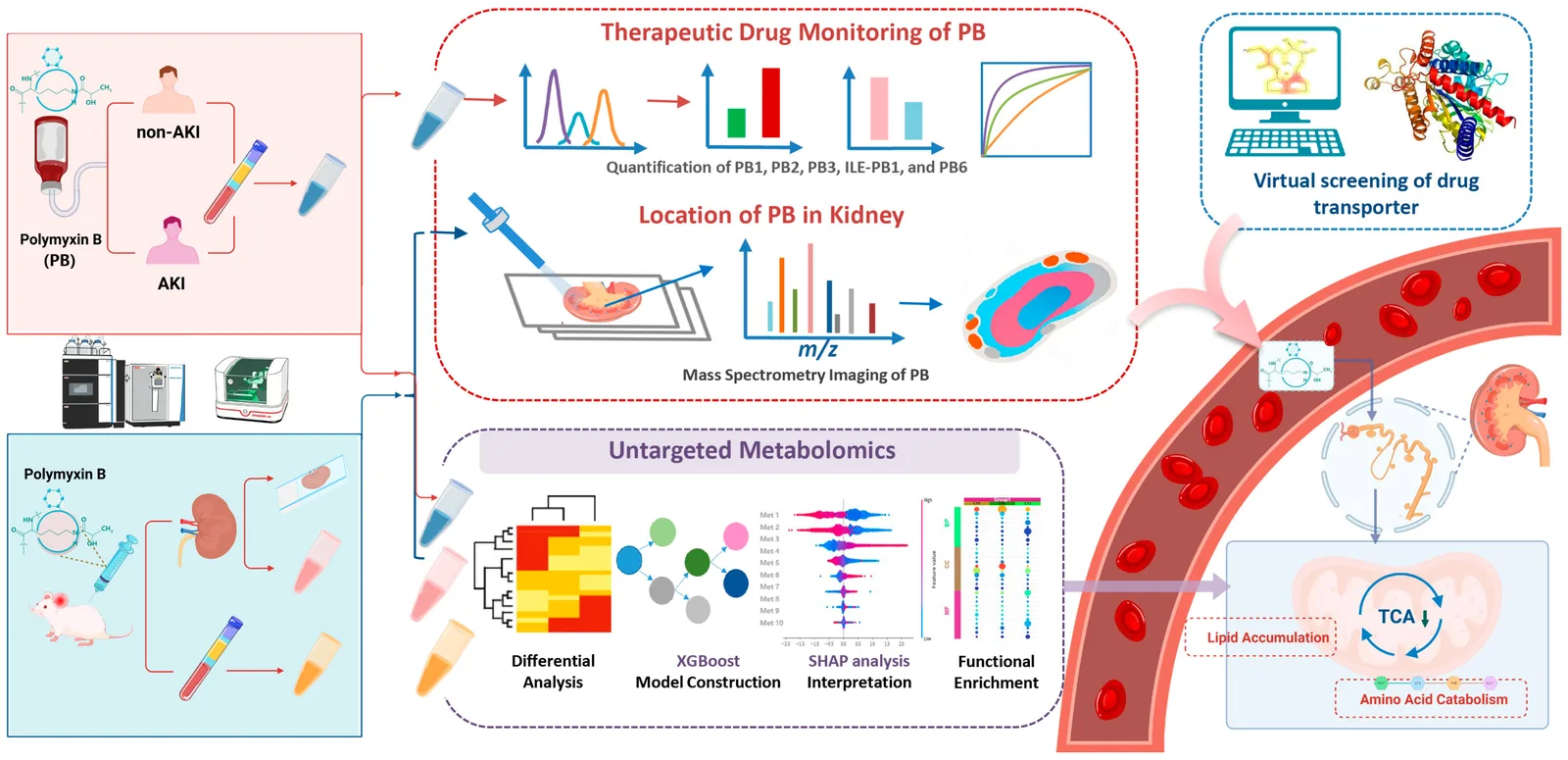
Schematic overview of the investigation of polymyxin B (PB)-associated acute kidney injury (AKI) using ultra-performance liquid chromatography coupled with high-resolution Orbitrap mass spectrometry (UPLC-HRMS), mass spectrometry imaging (MSI), and untargeted metabolomics. PB1, polymyxin B1; PB2, polymyxin B2; PB3, polymyxin B3; ILE-PB1, isoleucine-polymyxin B1; PB6, polymyxin B6; TCA, tricarboxylic acid cycle.

The current study was designed to optimize TDM parameters, identify biomarkers, and investigate the metabolic mechanisms of PB-induced AKI through quantitative analysis, mass spectrometry imaging, untargeted metabolomics, and machine learning. First, PB1, PB2, PB3, ILE-PB1, and PB6 were quantified in plasma from non-AKI and AKI patients by ultra-performance liquid chromatography coupled with high-resolution mass spectrometry (UPLC-HRMS) to evaluate TDM indicators. Next, airflow-assisted desorption electrospray ionization-mass spectrometry imaging (AFADESI-MSI) was used to map the localization and accumulation of PB in rat kidneys, and virtual screening was applied to identify targets mediating PB accumulation in renal tubules. Untargeted metabolomics was then performed on plasma from patients and on rat plasma and kidney samples to profile metabolic alterations. These metabolic changes were cross-validated, and the key pathways underlying PB-induced AKI were investigated. Finally, using machine learning algorithms, we developed a high-performance classifier based on 10 metabolites that may enable more accurate identification of patients with PB-associated AKI (see Figure 1 for a schematic representation of the research strategy). It is important to note the two-stage design of the present study. Human plasma samples from a clinical cohort are used primarily to define the diagnostic problem. By contrast, the mechanistic conclusions are derived principally from a rat model of PB-associated AKI and are then cross-checked, rather than proved, against the human metabolic data.

## 2. Results

### 2.1. A PB Component-Based Model Enables Precise Nephrotoxicity Risk Stratification Beyond Conventional TDM Parameters

**Figure 2:**
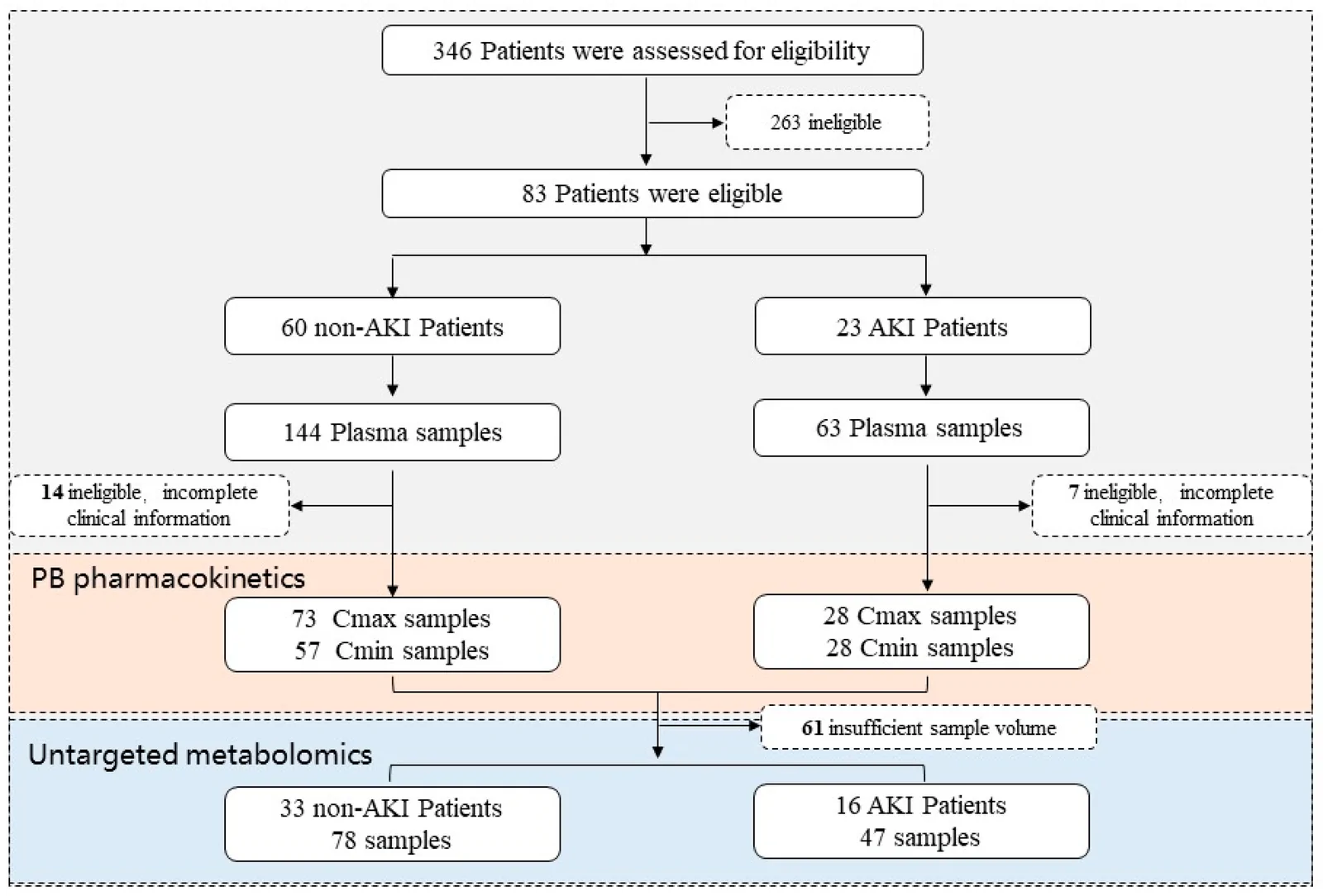
Patient selection flowchart. C_max_, peak concentration; C_min_, trough concentration.

Between April 2023 and September 2025, 346 patients were screened. After 263 patients were excluded, 83 patients were included in the study: 60 in the non-AKI group and 23 in the AKI group (Figure 2). The demographic and clinical characteristics of these patients are summarized in Table 1. No significant differences were observed between the two groups in age, sex, BMI, PB concentration distribution, or mortality.

**Figure 3:**
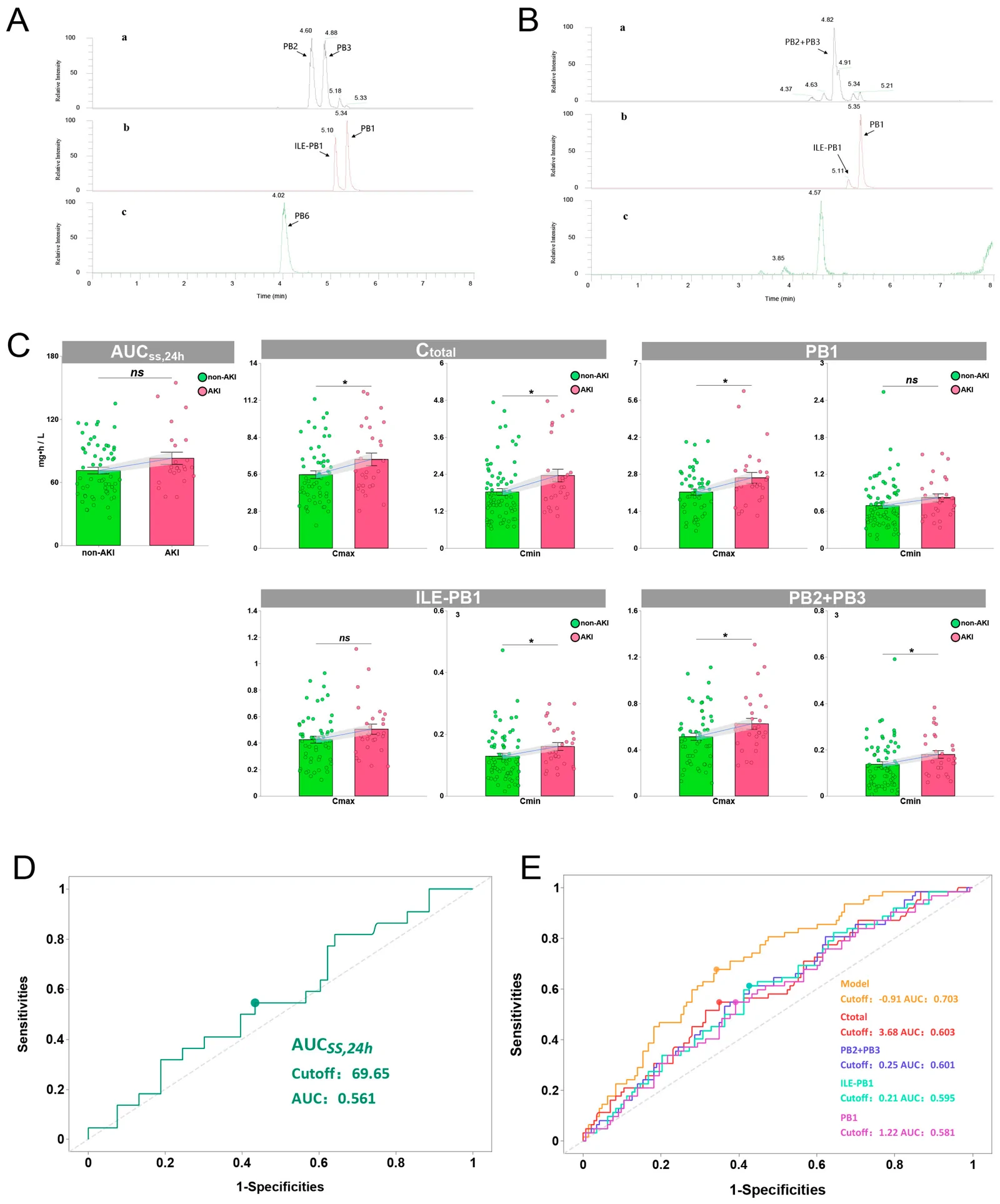
Identification of candidate parameters for therapeutic drug monitoring of polymyxin B (PB). (**A**) Representative UPLC-HRMS chromatograms of a blank plasma sample spiked with PB1, PB2, PB3, ILE-PB1, or PB6. (**B**) Representative UPLC-HRMS chromatograms of a typical patient plasma sample. (**C**) Comparison of AUC_ss,24h_, C_total_, PB1, ILE-PB1, and PB2 + PB3 levels between the non-AKI and AKI groups. Data are presented as the mean ± SD. Statistical differences were determined using a two-sided Wilcoxon rank-sum test (*, *p* < 0.05; ns, not significant). (**D**,**E**) Receiver operating characteristic (ROC) curves for AUC_ss,24h_, C_total_, PB1, ILE-PB1, PB2 + PB3, and a novel multivariate logistic regression model. PB1, polymyxin B1; PB2, polymyxin B2; PB3, polymyxin B3; ILE-PB1, isoleucine-polymyxin B1; PB6, polymyxin B6; AUC_ss,24h_, area under the concentration-time curve within 24 h; C_total_, total plasma concentration of PB; PB2 + PB3, sum of PB2 and PB3 concentrations; SD, standard deviation.

PB1, ILE-PB1, PB2, PB3, and PB6 were quantified by UPLC-HRMS. The total ion chromatograms (TICs) of the reference standards are shown in Figure 3A, and those of the PB components in patient plasma are shown in Figure 3B. Because PB2 and PB3 were chromatographically unresolved, they were quantified together. PB6 was present at nearly undetectable levels and was therefore excluded from the quantitative analysis. No significant between-group difference was observed in steady-state AUC_ss,24h_, the guideline-recommended TDM metric (Figure 3C). Significant intergroup differences were observed in trough concentration (C_min_) and C_max_ for the total PB components (C_total_) and the combination of PB2 and PB3. The concentrations of individual components also differed significantly between groups, including the C_max_ of PB1 and the C_min_ of ILE-PB1.

The discriminatory ability of PB components for AKI was assessed using ROC analysis and quantified by the area under the curve (AUC). Both AUC_ss,24h_ (ROC-AUC = 0.561, 95% confidence interval: 0.419–0.704; Figure 3D) and C_total_ (ROC-AUC = 0.603, 95% confidence interval: 0.519–0.687; Figure 3E) showed poor performance in distinguishing patients with AKI from those without AKI. Similarly, the AUCs for PB1, ILE-PB1, and PB2 + PB3 were all < 0.6. We then developed a multivariate logistic regression model incorporating these PB components. The model equation was P(AKI) = −6.76 × PB1 + 28.87 × ILE-PB1 + 6.62 × (PB2 + PB3) − 1.07. This model achieved an ROC-AUC of 0.703 (95% confidence interval: 0.633–0.781) for distinguishing PB-associated AKI, with intercept −1.07; coefficients: ILE_PB1 28.87, PB1 −6.76, PB2 + PB3 6.618; ORs rescaled to ng/mL for interpretability; Hosmer–Lemeshow *p* = 0.342 (Figure 3E).

### 2.2. P-glycoprotein (P-gp)-Mediated Transport as a Potential Mechanism for PB Tubular Accumulation

**Figure 4:**
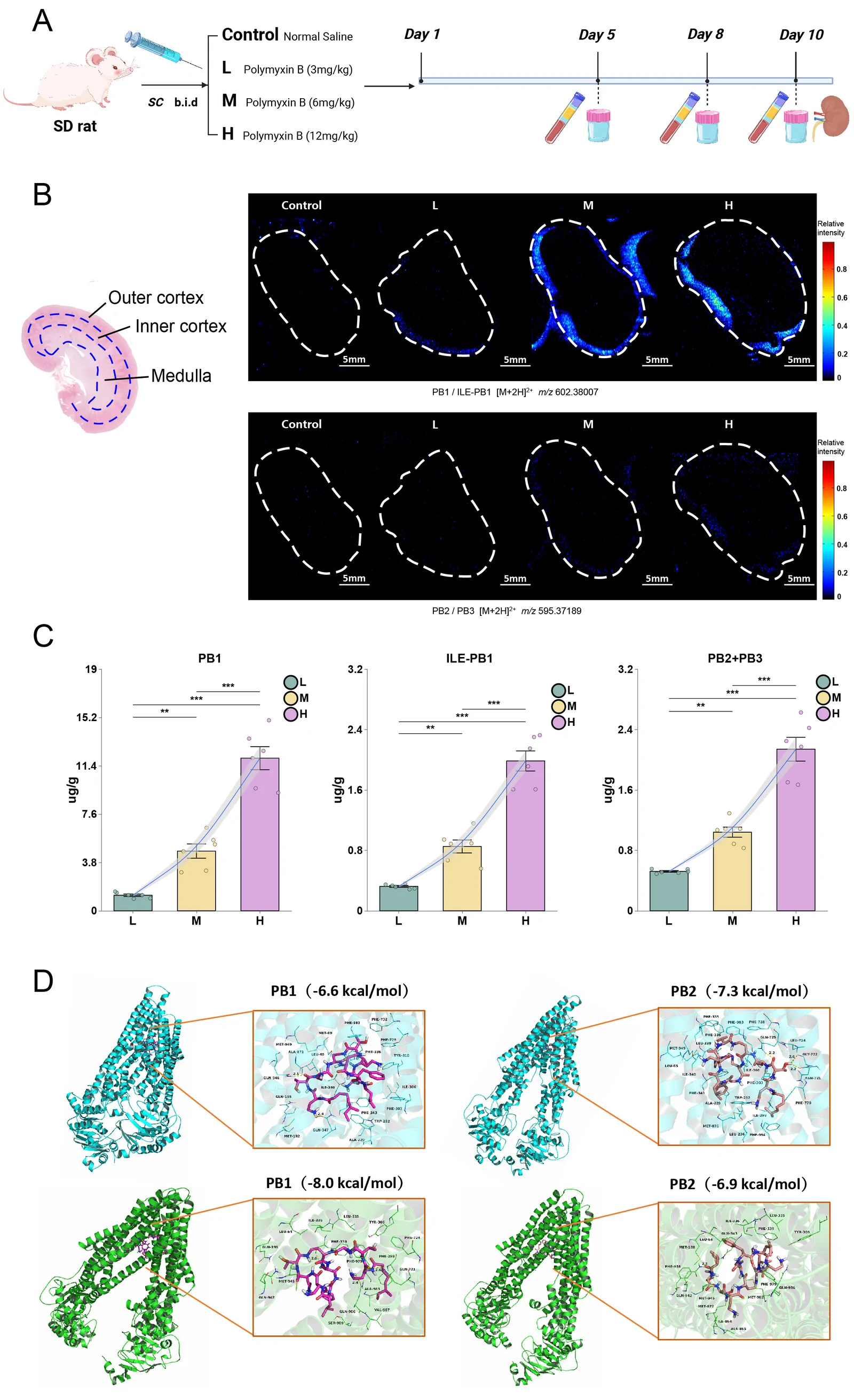
Renal accumulation of polymyxin B (PB) in treated rats. (**A**) Schematic of the PB-induced acute kidney injury (AKI) model. (**B**) Accumulation of PB1, ILE-PB1, PB2, and PB3 in the outer renal cortex after repeated dosing. (**C**) Quantification of PB components in kidney tissue. Data are presented as the mean ± SD (*n* = 12). Statistical differences were determined using Student’s *t* test (**, *p* < 0.01; ***, *p* < 0.001). (**D**) Molecular docking of PB1 and PB2 with P-glycoprotein (P-gp). Human P-gp is shown in blue, and rat P-gp is shown in green. PB1, polymyxin B1; PB2, polymyxin B2; PB3, polymyxin B3; ILE-PB1, isoleucine-polymyxin B1.

Dose-dependent increases in the renal concentrations of PB1, ILE-PB1, PB2, and PB3 were observed in rats administered 3 (low, L), 6 (medium, M), or 12 (high, H) mg/kg PB compared with control rats (Figure 4A,C). Each renal coronal section was divided into five anatomical and physiological regions: the whole kidney, outer cortex, inner cortex, medulla, and pelvis. MSI detected PB components and visualized their accumulation in the outer cortex (Figure 4B). Renal PB accumulation is considered a key driver of AKI, and its underlying mechanisms therefore require elucidation.

Drug disposition and accumulation are governed by the interplay between metabolizing enzymes and membrane transporters. The toxicity endpoints of the PB components were predicted using ACD/Tox Suite 2.95 and ACD/ADME Suite 5.0 (Table S2). All four components (PB1, PB2, PB3, and ILE-PB1) were predicted to be substrates of P-glycoprotein (P-gp). The renal cortex, where PB components accumulated (Figure 4B), spatially corresponds to the region of high P-gp expression on the apical membrane of the tubular epithelium [19]. This spatial correspondence is consistent with a potential interaction between PB components and renal P-gp.

In molecular docking studies, PB1 and PB2 exhibited high-affinity binding to both human and rat P-gp (Figure 4D). For human P-gp, the binding energies were −6.6 kcal/mol (PB1) and −7.3 kcal/mol (PB2); for rat P-gp, the binding energies were −8.0 kcal/mol (PB1) and −6.9 kcal/mol (PB2). PB1 and PB2 bound to hydrophobic cavities formed by multiple residues (Table S3). This conserved high-affinity binding suggests that PB1 and PB2 may function as P-gp substrates.

### 2.3. Progressive Deterioration of Kidney Function and Structural Damage in Rats with PB-Induced AKI

**Figure 5:**
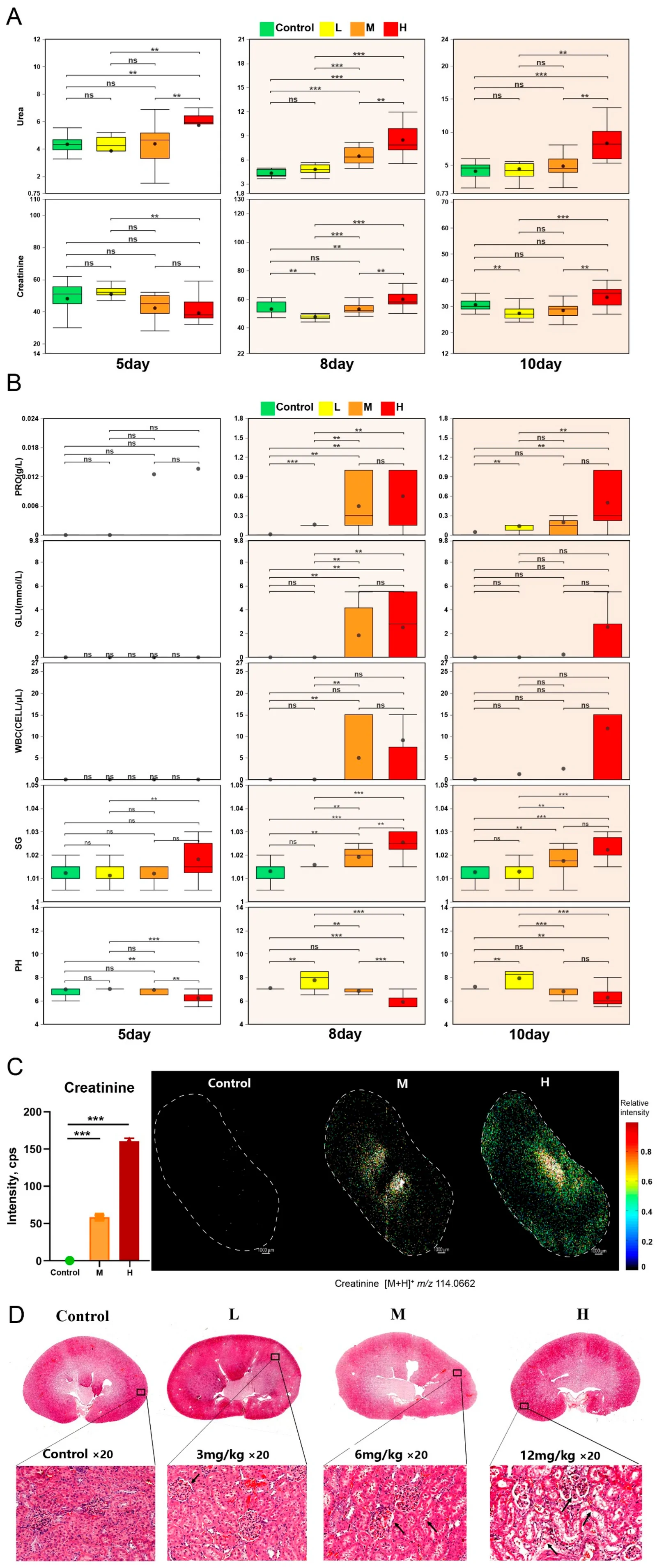
Progressive decline in kidney function in rats with polymyxin B (PB)-induced acute kidney injury (AKI). (**A**) Changes in urea and serum creatinine levels at different time points after PB injection (*n =* 12). (**B**) Changes in urinary protein (PRO), glucose (GLU), white blood cell (WBC) count, specific gravity (SG), and pH over time (*n =* 12). (**C**) Spatial distribution of creatinine in kidney tissue determined by mass spectrometry imaging. (**D**) Representative hematoxylin and eosin (H&E)-stained kidney sections from control rats and rats treated with low (L, 3 mg/kg), medium (M, 6 mg/kg), or high (H, 12 mg/kg) doses of PB. In (**A**–**C**), significant differences were determined using Student’s *t* test (**, *p* < 0.01; ***, *p* < 0.001; ns, not significant).

Rats with PB-induced AKI exhibited progressive weight loss (*p* < 0.01; Table S4) and an increased kidney index by day 10, consistent with renal edema or compensatory hypertrophy. Renal function markers exhibited both time- and dose-dependent patterns. Kidney function was severely impaired, with urea and serum creatinine levels markedly elevated on days 8 and 10 (Figure 5A, Table S5). The presence of urinary protein (PRO) and glucose (GLU) on days 8 and 10 indicated severe tubular reabsorptive dysfunction (Figure 5B, Table S7). Increased urine specific gravity (SG) was coupled with decreased pH on days 5, 8, and 10 (Figure 5B, Table S6).

Furthermore, compared with the control group, the H group had significantly lower levels of alkaline phosphatase (ALP), albumin (Alb), and total protein (TP) and a higher level of total cholesterol (TC) (Figure S1, Table S5). Concurrently, an increased total white blood cell (WBC) count and decreased red blood cell (RBC) count and hemoglobin (HGB) level were observed (Figure S2, Table S7). These alterations are consistent with the multisystem effects of PB-induced AKI, including renal dysfunction, altered protein metabolism, and systemic inflammatory responses.

To assess in situ renal injury, molecular profiling was performed by MALDI-MSI on frozen kidney sections (Figure 5C). Imaging analysis revealed a significant dose-dependent increase in tissue creatinine signal intensity. H&E staining showed intact glomeruli with clear capillary loops and well-organized tubules in the controls (Figure 5D). In contrast, PB-treated rats exhibited grade 1 lesions (semiquantitative score [SQS] = +1), characterized by tubular dilation, degeneration, and marked lipid droplet accumulation. These pathological changes indicate that PB causes progressive tubular lesions in rats.

### 2.4. Metabolic Profiling of Control and PB-Treated Rats

**Figure 6:**
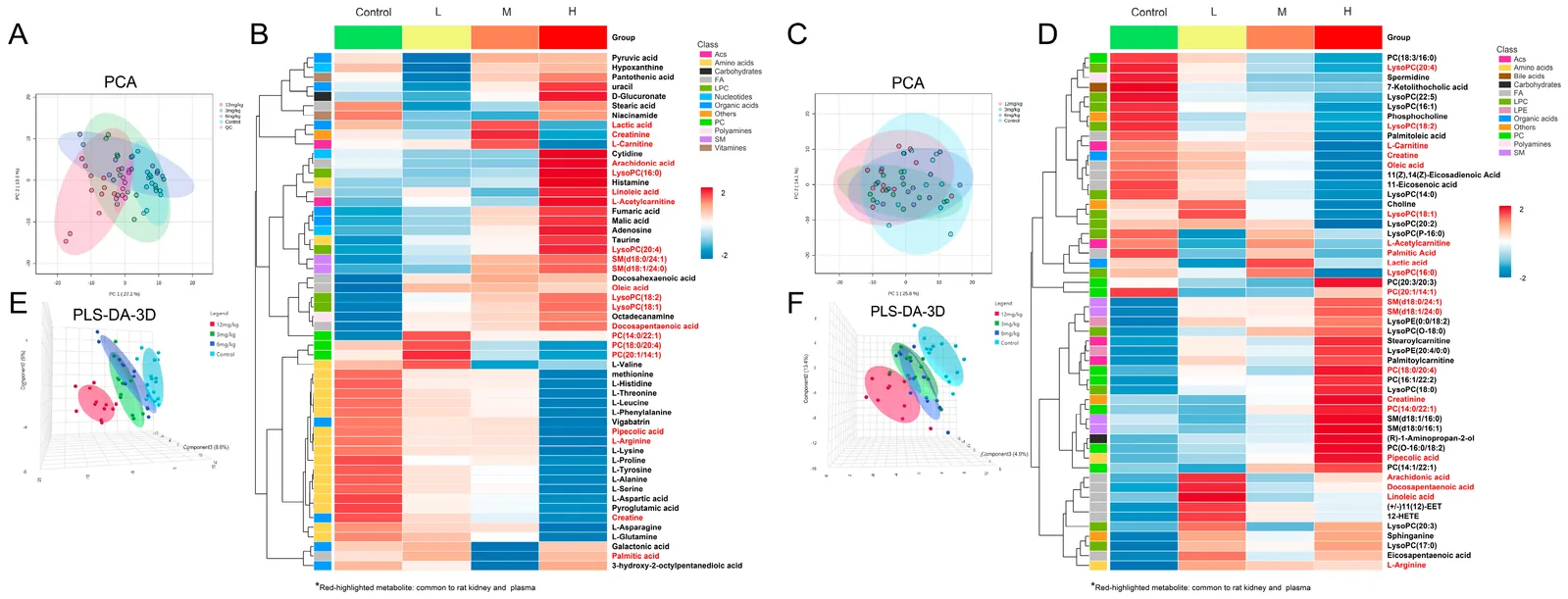
Multivariate statistical analysis of untargeted metabolomics in rats. (**A**) Principal component analysis (PCA) score plot of kidney metabolomics data from control rats and polymyxin B (PB)-treated rats administered low (L, 3 mg/kg), medium (M, 6 mg/kg), or high (H, 12 mg/kg) doses. (**B**) Heatmap of significantly altered kidney metabolites between the control and PB-treated groups (*p* < 0.05 according to the Kruskal–Wallis test). (**C**) PCA score plot of plasma metabolomics data from control and PB-treated rats. (**D**) Heatmap of significantly altered plasma metabolites between the control and PB-treated groups (*p* < 0.05 according to the Kruskal–Wallis test). (**E**,**F**) Partial least squares discriminant analysis (PLS-DA) of kidney and plasma metabolomics data from control and PB-treated rats.

Changes in the metabolic profiles of rat kidney and plasma samples were characterized using principal component analysis (PCA) and partial least squares discriminant analysis (PLS-DA); the model parameters are detailed in Table S8. The PCA score plot showed separate clustering of kidney samples from the control and PB groups (Figure 6A), whereas the PLS-DA score plot showed more distinct clustering between the two groups (Figure 6E). Similarly, plasma samples were completely separated in both models (Figure 6C,F).

A total of 54 differentially abundant metabolites were identified in the rat kidney (VIP > 1) and categorized into 12 classes based on their chemical structures and metabolic pathways (Figure 6B and Table S9). Among these, 28 metabolites were increased, including 6 fatty acids (FAs), 6 organic acids (OAs), and 4 lysophosphatidylcholines (LPCs), as well as sphingomyelins (SMs), nucleotides, and amino acids. Of the remaining 26 decreased metabolites, 17 were amino acids, 3 were OAs, and 2 were phosphatidylcholines (PCs). Similarly, 53 metabolites were altered in rat plasma (29 increased and 24 decreased), with notable changes in FAs, phospholipids (PCs, LPCs, and SMs), acylcarnitines (ACs), and amino acids (Figure 6D and Table S10). We further identified 21 differentially abundant metabolites common to both plasma and kidney samples (highlighted in red in Figure 6B,D). The major shared classes included 5 FAs, 4 LPCs, 3 PCs, 2 SMs, 2 ACs, and 2 OAs. Arginine, creatine, and creatinine were also altered in both matrices.

Collectively, these findings indicate that PB induces broad metabolic perturbations in lipids, amino acids, and carbohydrates.

### 2.5. A Machine Learning-Based Metabolomic Classifier Distinguishes PB-Associated AKI from Non-AKI

**Figure 7:**
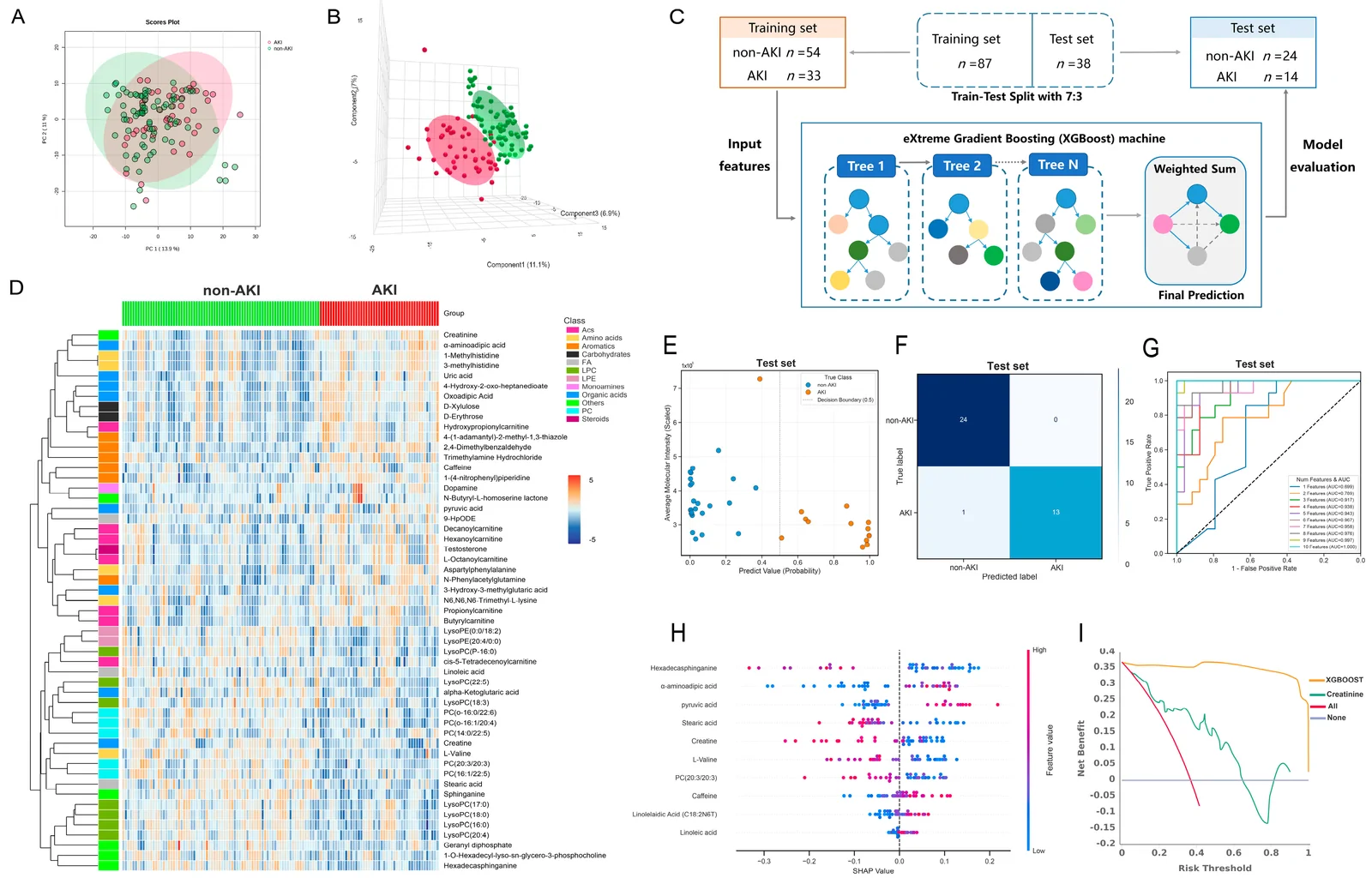
Machine learning-derived discrimination model based on the plasma metabolome for distinguishing patients with polymyxin B (PB)-associated acute kidney injury (AKI) from those without AKI. (**A**) Principal component analysis (PCA) score plot of plasma metabolomics data from non-AKI patients and patients with PB-associated AKI. (**B**) Partial least squares discriminant analysis (PLS-DA) of plasma metabolomics data from non-AKI patients and patients with PB-associated AKI. (**C**) Design of the modeling workflow. The eXtreme Gradient Boosting (XGBoost) algorithm was used for feature selection and model training in the discovery set. (**D**) Heatmap of plasma metabolite alterations between the non-AKI and PB-associated AKI groups. Significantly altered metabolites were identified using the Kruskal–Wallis test (*p* < 0.05). (**E**) Scatter plot showing the prediction probabilities generated by the XGBoost model to distinguish patients without AKI from those with PB- associated AKI in the test set. The horizontal dashed line represents the decision boundary at a probability of 0.5. (**F**) Confusion matrix derived from the XGBoost model for distinguishing patients without AKI from those with PB-associated AKI in the test set. (**G**) Receiver operating characteristic (ROC) curve and area under the curve (AUC) for identifying patients with PB-associated AKI in the test set. (**H**) SHapley Additive exPlanations (SHAP) diagram. The 6 features effectively identify PB-associated AKI. (**I**) Decision curve analysis (DCA) in the test set. Net benefit is plotted across a range of threshold probabilities, comparing the model with creatinine, treat-all, and treat-none strategies.

Distinct metabolic perturbations were also observed in the plasma metabolic profiles of patients with and without PB-associated AKI, as revealed by PCA and PLS-DA (Figure 7A,B). A total of 53 metabolites (VIP > 1, *p* < 0.05) were significantly altered, with 29 increased and 24 decreased in patients with AKI (Figure 7D and Table S11). The increased metabolites were mainly ACs, OAs, aromatic compounds, amino acids, and creatinine. The decreased metabolites were primarily lysophospholipids (7 LPCs and 2 LPEs), PCs (5), FAs (2), and creatine.

The altered metabolic profiles were subsequently subjected to XGBoost-based feature selection before model construction, yielding 10 optimal metabolites ranked by gain: spermine, sphinganine, hexadecasphinganine, pg(18:0/18:0), butyrylcarnitine, pyridoxine, etc. (Figure S3). According to the gains, six endogenous metabolites were screened from them for model construction using the XGBoost algorithm (Figure 7C). The model was trained to distinguish patients with AKI from those without AKI and was validated using a test set. In the test set, the model achieved an accuracy of 0.95 (95% CI: 0.82–0.99), AUC 1.00 (95% CI: 1.00–1.00), sensitivity 0.92 (95% CI: 0.73–0.99), specificity 1.00 (95% CI: 0.77–1.00), precision 1.00 (95% CI: 0.85–1.00), F1 score 0.96. (Figure 7E–G). The model demonstrated higher discriminatory performance compared with the conventional renal function indicator creatinine (AUC = 0.740) (Figure S4). The SHAP feature-importance ranking showed that hexadecasphinganine contributed most to the model predictions, followed by spermine, sphinganine, and hexadecasphinganine; butyrylcarnitine and pyridoxine made a smaller contribution (Figure 7H). We subsequently conducted decision curve analysis (DCA) to assess the clinical utility of the model in the test set. Across a threshold probability range of 0.1–1.0, the results indicated a net benefit from model-guided intervention in patients with AKI (Figure 7I).

Notably, creatine was an overlapping metabolite across human plasma, rat kidney, and rat plasma. Plasma creatine was negatively correlated with creatinine in patients with AKI (r = −0.47, *p* = 0.001) but positively correlated with creatinine in non-AKI controls (r = 0.24, *p* < 0.05; Figure S5A). In addition, a dose-dependent decrease was observed in rat kidney and plasma samples (Figure S5B). This reduction was already apparent at the lowest dose, corresponding to mild kidney injury. In contrast, plasma creatinine increased only with severe injury (M and H groups) and decreased in the L group. Consistently, AFADESI-MSI confirmed a dose-dependent decrease in renal creatine signal intensity (Figure S5C). Supporting this finding, Locci et al. reported an 86.52-fold increase in rat urinary creatine within the first 24 h of PB-induced AKI [13]. Taken together, these data suggest that creatine is a promising diagnostic marker for PB-associated AKI and may complement creatinine.

### 2.6. Metabolic Pathway Analysis

**Figure 8:**
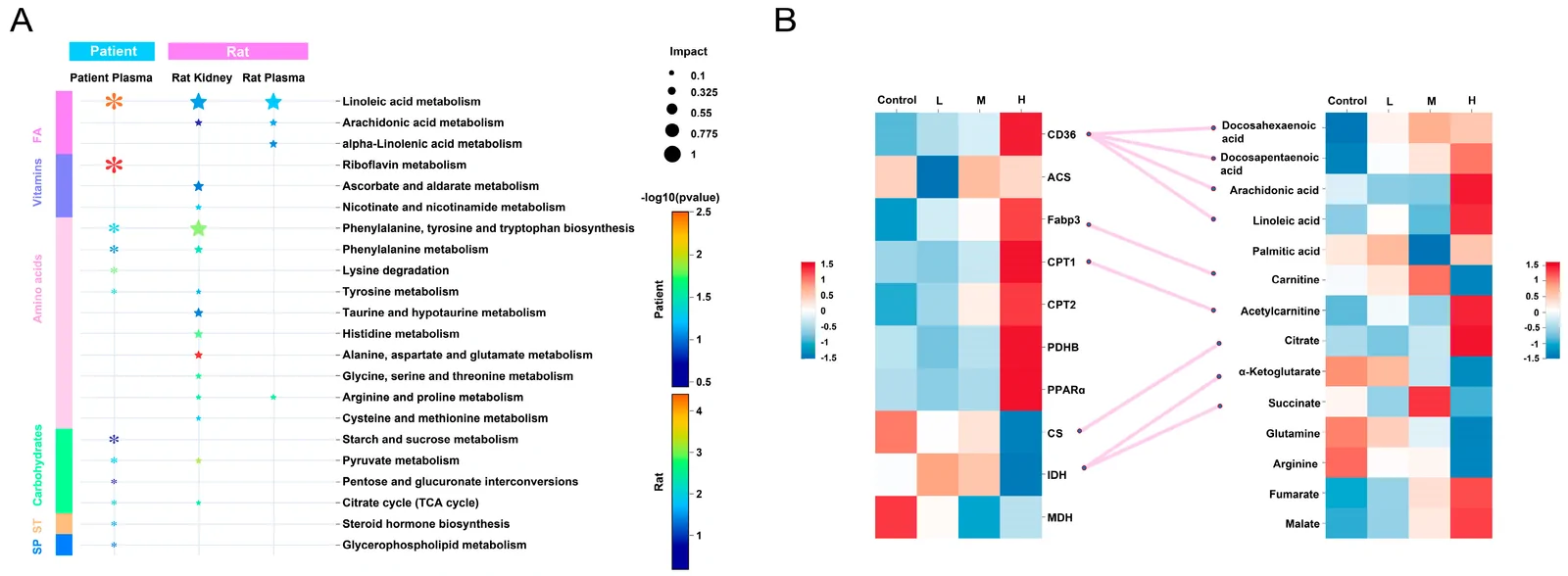
(**A**) Kyoto Encyclopedia of Genes and Genomes (KEGG) metabolic pathways enriched with significantly altered metabolites in non-AKI versus PB-induced AKI patients and in control versus PB-treated rats. Pathway enrichment was assessed using a one-sided Fisher’s exact test with Benjamini-Hochberg (BH) false discovery rate (FDR) correction (FDR < 0.05). (**B**) Heatmap of altered kidney metabolites involved in fatty acid metabolism and the TCA cycle in PB-treated versus control rats.

Kyoto Encyclopedia of Genes and Genomes (KEGG) pathway analysis revealed disruption of multiple metabolic pathways (pathway impact > 0.1) in patients with AKI compared with controls, with linoleic acid metabolism showing the most significant enrichment (Figure 8A). Consistent with the clinical data, rat kidneys exhibited marked disturbances in several metabolic pathways, mainly linoleic acid metabolism, arachidonic acid metabolism, and phenylalanine, tyrosine, and tryptophan biosynthesis (Figure 8A). Linoleic acid metabolism was also the most perturbed pathway in rat plasma, suggesting that disruption of FA metabolism is involved in the pathogenesis of PB-induced AKI (Figure 8A).

### 2.7. Perturbation of Fatty Acid Metabolism and the TCA Cycle in PB-Induced AKI

**Figure 9:**
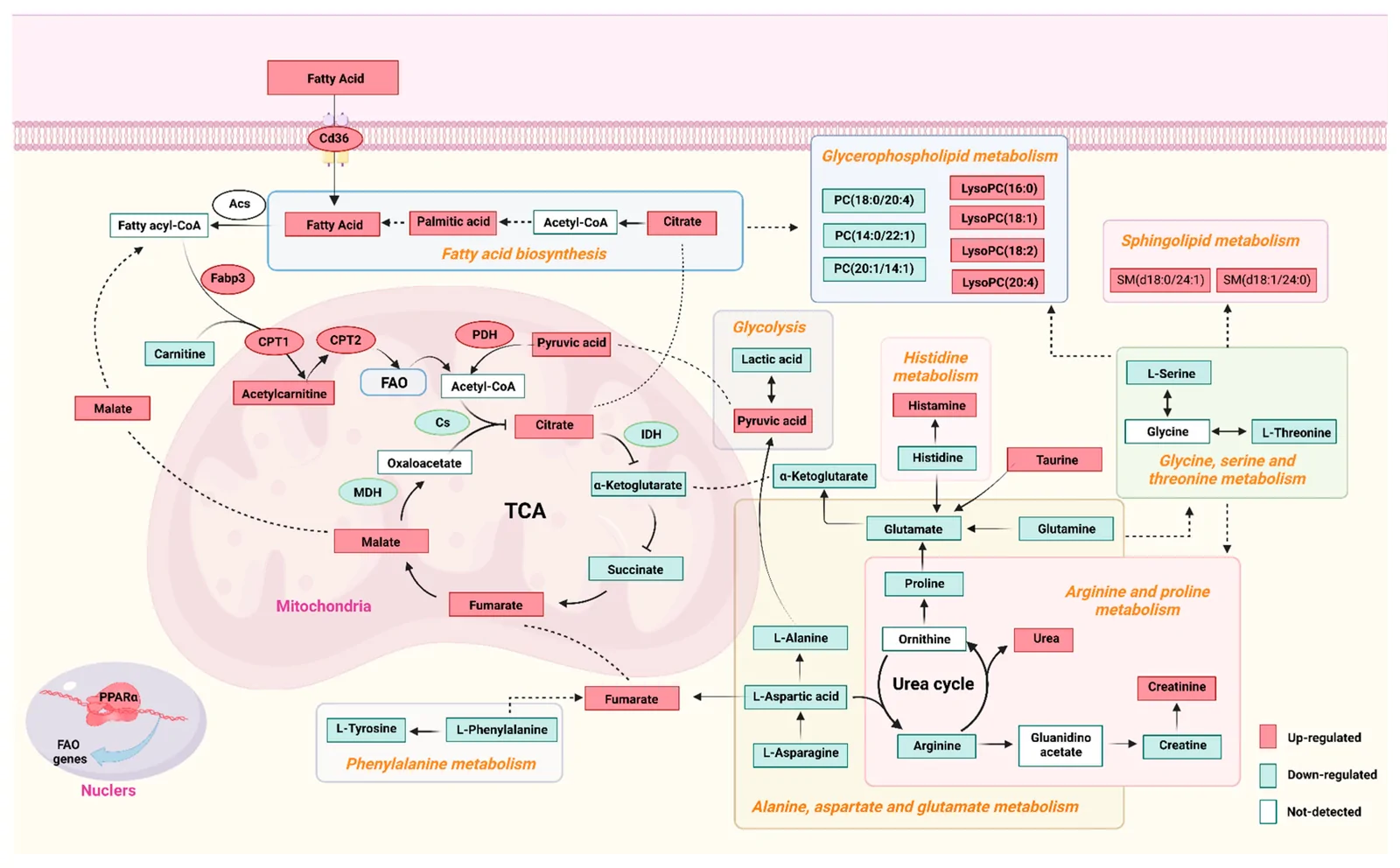
Schematic overview of the metabolic mechanisms underlying polymyxin B (PB)-induced acute kidney injury (AKI). ACS, acyl-CoA synthetase; CD36, cluster of differentiation 36; CPT1, carnitine palmitoyltransferase 1; CPT2, carnitine palmitoyltransferase 2; CS, citrate synthase; FAO, fatty acid oxidation; FABP3, fatty acid-binding protein 3; IDH, isocitrate dehydrogenase; MDH, malate dehydrogenase; PDH, pyruvate dehydrogenase; PPARα, peroxisome proliferator-activated receptor-α; TCA, tricarboxylic acid cycle.

To further explore the effects of PB-induced AKI on renal energy metabolism, we assessed alterations in FA metabolism and TCA cycle pathways by analyzing 14 related metabolites and 10 metabolism-associated proteins (Figure 8B); a summary scheme is shown in Figure 9. RT-qPCR analysis revealed increased mRNA transcript abundance of CD36, a key fatty acid transporter, consistent with increased renal fatty acid uptake. This change was accompanied by the progressive accumulation of docosahexaenoic acid (DHA) and docosapentaenoic acid (DPA) in the kidney. Arachidonic acid, linoleic acid, and palmitic acid were also markedly elevated in the high-dose (H) group. Despite unchanged acyl-CoA synthetase (ACS) expression, suggesting limited fatty acid activation, a coordinated mitochondrial adaptive response mediated by peroxisome proliferator-activated receptor-α (PPARα) was observed. This adaptive reprogramming involved upregulation of fatty acid-binding protein 3 (FABP3), carnitine palmitoyltransferase 1 (CPT1), and CPT2 [20], thereby facilitating cytosolic fatty acyl-carnitine shuttling and mitochondrial import.

Acetyl-CoA generation was increased through two inputs: fatty acid β-oxidation and increased pyruvate oxidation through upregulation of pyruvate dehydrogenase (PDH). However, PB induced profound renal metabolic reprogramming characterized by downregulation of the TCA cycle (Figure 8B, Tables S12 and S13). First, despite downregulated citrate synthase (CS) expression, residual enzymatic activity sustained citrate production. Concurrently, severe downstream inhibition created a metabolic bottleneck: downregulation of isocitrate dehydrogenase (IDH) impaired α-ketoglutarate (α-KG) synthesis, an effect compounded by depletion of the anaplerotic precursors glutamine and arginine, which normally replenish α-KG through glutaminolysis. Second, suppression of malate dehydrogenase (MDH) caused malate accumulation, whereas impaired fumarate conversion further disrupted cycle continuity. These sequential blockades prevented efficient acetyl-CoA oxidation, creating a futile metabolic cycle in which increased substrate supply converged with impaired catabolic capacity. The accumulated citrate was likely exported to the cytosol, where it served as a lipogenic substrate.

Together, these results were consistent with the lipid droplet accumulation observed in H&E-stained kidney tissue sections, suggesting that perturbations in FA metabolism and the TCA cycle contribute to the pathogenesis of PB-induced AKI.

## 3. Discussion

In this study, we successfully developed two models: a PB TDM model and an AI-based diagnostic model for PB-associated AKI. Mechanistically, we found that high PB concentrations might saturate P-gp, leading to PB accumulation in renal tubules and subsequent metabolic dysregulation, manifested as upregulation of FA pathways and downregulation of the TCA cycle. To the best of our knowledge, this is the most comprehensive study to date to investigate the biomarkers and mechanisms of PB-induced nephrotoxicity.

Currently, TDM is recommended for PB dose adjustment to reduce both antimicrobial resistance and adverse reactions, particularly nephrotoxicity [16,17]. Although AUC_ss,24h_ has been established as the TDM parameter by international consensus [17], the supporting evidence remains limited. Only two studies cited in the consensus proposed specific pharmacokinetic thresholds [21,22]. However, one relied solely on simulation algorithms, and neither addressed the distinct pharmacokinetic profiles of individual PB components. Notably, our multivariate logistic regression model exhibited better discriminatory performance (ROC-AUC = 0.703) compared with AUC_ss,24h_ (ROC-AUC = 0.561). Consistent with our findings, a recent study demonstrated that a model combining C_max_ (PB1), baseline BUN, and hypertension is a robust predictor of PB-induced AKI [18]. Overall, the novel model may complement existing TDM frameworks by enabling accurate identification of patients at elevated risk of nephrotoxicity and thereby facilitating individualized dose optimization.

Serum creatinine is not an optimal biomarker because kidney excretory function must decline by at least 60% before serum creatinine increases [5]. Notably, an XGBoost model based on six metabolites was developed to distinguish patients with PB-associated AKI from those without AKI. This model demonstrated excellent performance in the test set, indicating diagnostic ability. SHAP analysis subsequently provided a transparent explanation of how individual features influenced the prediction outcomes. DCA demonstrated that the XGBoost model provided a greater net clinical benefit than creatinine across a wide range of threshold probabilities. To date, only one machine learning model has been used to predict PB-associated nephrotoxicity [23]. Several important differences exist between that study and ours. First, the model inputs were fundamentally different: the previous model relied on routine clinical parameters, whereas our model was based on plasma metabolomics data, which provide more dynamic, novel, and sensitive biomarkers. Second, the two studies differed in their scientific focus. The previous study focused mainly on comparing the nephrotoxicity of colistin with that of polymyxin B and did not assess model interpretability or clinical utility. In contrast, our study not only presented an exploratory model with internal validation but also analyzed its interpretability and net clinical benefit in depth.

Notably, the model identified creatine as a key biomarker that diagnostics AKI. Because the kidney is the primary organ involved in the first step of creatine synthesis (the formation of guanidinoacetate) [24], creatine levels are readily affected when renal function is impaired. Serum creatine levels have been reported to increase significantly at 24 h during the early stage of sepsis-associated AKI [25]. Urinary creatine levels were elevated in mice with aristolochic acid-induced AKI [26], potentially reflecting oxidative modification of renal mitochondria or creatine kinase dysfunction [27]. Our findings demonstrate the value of metabolomics and machine learning for biomarker discovery and suggest that these approaches may enable more precise detection of PB-associated AKI.

Mechanistically, PB nephrotoxicity is initiated by extensive PB accumulation in the proximal tubules, a process that occurs predominantly within the renal cortex. In the absence of definitive transporter experiments, and based on molecular docking studies, we speculated that PB might be a substrate of P-gp, consistent with previous reports characterizing PB as a substrate of ATP-binding cassette transporters in bacterial and mammalian systems [28,29]. Under physiological conditions, P-gp-mediated apical efflux protects the tubular epithelium from xenobiotic injury. However, at elevated PB doses, transporter saturation compromises this detoxification mechanism. Accordingly, we observed dose-dependent increases in renal cortical PB concentrations, indicating that the intratubular drug load exceeded the P-gp efflux capacity. Notably, ABCB1 (encoding P-gp) is subject to genetic polymorphisms, epigenetic regulation, and drug–drug interactions; reduced P-gp capacity would be predicted to lower the threshold for tubular PB accumulation and thus amplify nephrotoxicity at a given plasma exposure, whereas higher transporter activity may attenuate it.

Following PB accumulation in the renal tubules, metabolic disturbances occur. We found that FA metabolism was severely disrupted across all analyzed datasets (human plasma, rat kidney, and rat plasma), resulting in renal FA accumulation. These findings align with emerging evidence that patients with AKI exhibit substantial disruption of fatty acid oxidation (FAO) and abnormal FA metabolism [20]. These alterations ultimately lead to ATP depletion and lipid peroxidation, which are central to AKI pathophysiology. Profiling of rat renal FAs revealed significant increases in saturated FAs (palmitic and stearic acids), monounsaturated FAs (oleic acid), and polyunsaturated FAs (linoleic acid, arachidonic acid, DHA, and DPA). Because palmitic acid is the primary product of de novo lipogenesis [30], its accumulation may reflect enhanced uptake or impaired catabolism rather than local synthesis. Conversely, circulating levels of linoleic and oleic acids were decreased in both humans and rats, suggesting enhanced renal FA extraction. This process may be facilitated by CD36 upregulation [8].

Carnitine plays a vital role in cellular energy distribution by mediating the transport of long-chain FAs across the inner mitochondrial membrane. In this process, fatty acyl-CoA and carnitine are conjugated by CPT1 to form long-chain acylcarnitines, which are subsequently converted back to fatty acyl-CoA by CPT2 for β-oxidation, generating acetyl-CoA to fuel the TCA cycle [20]. We observed an initial increase followed by a significant decrease in renal carnitine levels with increasing PB dosage, together with a consistent dose-dependent decrease in plasma carnitine levels. We propose that, at low doses, increased renal uptake from the circulation compensates for changes in tissue carnitine homeostasis. However, at higher doses, the progressively depleted circulating pool becomes insufficient to sustain renal uptake, leading to a marked decline in tissue carnitine levels. Similarly, carnitine deficiency has been implicated as a risk factor for methotrexate-mediated AKI [31], suggesting a shared metabolic vulnerability in drug-induced nephrotoxicity. Furthermore, impaired β-oxidation leads to acyl-CoA accumulation, which sequesters available carnitine as acylcarnitine [32]. Consistent with this mechanism, a dose-dependent increase in renal acetylcarnitine levels was observed after PB treatment. Collectively, these findings suggest that carnitine depletion impairs mitochondrial FA transport and subsequent β-oxidation, thereby exacerbating the energy deficit in PB-induced AKI.

The mitochondria-rich proximal convoluted tubule depends on β-oxidation for energy generation, through which FAs are converted to acetyl-CoA to fuel the TCA cycle [33]. PB-induced AKI has previously been shown to alter urinary TCA cycle intermediates in rats [13]. Consistent with these reports, we found that the key TCA cycle regulatory enzymes CS and MDH showed reduced expression. This metabolic blockade was further reflected by marked alterations in metabolite profiles, with significantly reduced levels of α-KG and succinate and increased accumulation of isocitrate, fumarate, and malate. Furthermore, creatine, a critical component of the creatine kinase-phosphocreatine circuit that couples ATP production to sites of consumption [31], was consistently depleted in rat kidney, rat plasma, and human plasma. Together with the evidence of TCA cycle inhibition, these findings support mitochondrial dysfunction as a central feature of PB-induced AKI.

Although FAs and TCA cycle intermediates are important energy sources for the kidney, amino acid metabolism is essential for maintaining renal nitrogen balance and systemic metabolic homeostasis. The kidney maintains balanced amino acid metabolism through synthesis, degradation, filtration, reabsorption, and excretion [34]. Reabsorbed amino acids serve as gluconeogenic substrates and, after deamination, replenish TCA cycle intermediates for oxidation. We observed severe disruption of amino acid metabolism in PB-induced AKI, as evidenced by 19 altered amino acids in rat kidneys, of which 2 were increased and 17 were decreased. Taurine showed the greatest increase, consistent with earlier observations of increased urinary taurine in rats with PB-induced AKI [13]. This elevation may be attributable to the cytoprotective role of taurine against oxidative stress through suppression of mitochondrial reactive oxygen species (ROS) generation [35]. Histamine was the second most upregulated metabolite, likely reflecting PB-induced mast cell degranulation [3]. The underlying mechanism may involve mast cell infiltration; mast cells are the principal source of histamine through the histidine decarboxylase-mediated conversion of histidine to histamine [36]. This pathway mechanistically links the observed depletion of renal histidine to the concurrent accumulation of histamine.

Reductions in aspartic acid, asparagine, and alanine, which feed into the TCA cycle and glycolysis, together with decreased phenylalanine, suggest impaired energy metabolism and disrupted cellular homeostasis [37]. In the proximal tubules, glutamine is metabolized to glutamate, which serves as a precursor of α-KG, an intermediate in the TCA cycle [38]. The significant depletion of renal glutamine and glutamate in our study was consistent with decreased urinary glutamine after PB treatment [13]. We speculate that accelerated catabolism of these amino acids reflects compensatory anaplerotic flux into the TCA cycle during PB-induced AKI.

The kidney is the primary site of arginine synthesis and is responsible for maintaining systemic arginine homeostasis. Arginine serves as a metabolic hub in the urea cycle and arginine-proline metabolism, acting as a direct precursor of urea, creatine, and nitric oxide and as an upstream metabolite of creatinine [27]. We observed significantly decreased levels of arginine, proline, and creatine and significantly increased levels of creatinine and urea in the kidneys of PB-treated rats. This metabolic pattern suggests enhanced flux through the creatine biosynthesis pathway and the urea cycle, a phenomenon also observed in ischemia–reperfusion-induced AKI [39].

Branched-chain amino acids (BCAAs), including leucine, valine, and isoleucine, are important energy sources for the kidney [40]. They are metabolized by branched-chain aminotransferases to generate branched-chain α-KG, which is then oxidatively decarboxylated by the branched-chain α-KG dehydrogenase complex, producing acyl-CoA derivatives that ultimately yield acetyl-CoA and/or succinyl-CoA for entry into the TCA cycle [41]. In the kidney, leucine and valine levels decreased significantly in a dose-dependent manner after PB administration (Figure 6B). Recent studies have reported reduced activity of BCAA catabolic enzymes in aristolochic acid-induced nephropathy and septic AKI [42,43]. This impairment of BCAA catabolism may compromise ATP production in PB-induced AKI, particularly given the concomitant disruption of FAO, because BCAA-derived acetyl-CoA and succinyl-CoA normally serve as critical anaplerotic substrates for the TCA cycle [44].

The metabolism of glycine, serine, and threonine contributes to the structural integrity of renal cell membranes through phospholipid synthesis (e.g., phosphatidylserine) and supports renal adaptation to lipid stress by modulating sphingolipid synthesis and the glutathione antioxidant system, thereby influencing injury and repair processes [45]. In this study, the decreased concentrations of serine and threonine, which are essential for maintaining protein structure and membrane integrity, suggest compromised cellular homeostasis.

Disorders of lipid metabolism lead to increased ROS production, oxidative stress, inflammation, and cell death [20]. In the PB-induced AKI groups, we observed aberrant metabolism of PC, LPC, and SM, manifested as decreased levels of two PC species and elevated levels of four LPC species in the kidney. PC depletion suggests impaired membrane integrity, whereas elevated LPC levels act as danger signals reflecting oxidative stress, inflammation, and apoptosis [46]. Moreover, SMs play important roles in cell structure, signaling, inflammation, and lipid transport during kidney injury [47]. In our study, two SMs were consistently increased in the kidney after PB exposure. Together, these metabolomics findings indicate that dysregulation of PC, LPC, and SM in the kidney contributes to impaired energy homeostasis, disrupted membrane integrity, and inflammation in PB-induced AKI.

This study has several notable strengths. The integration of multiple mass spectrometry techniques improved the accuracy of biomarker identification, whereas cross-species validation strengthened the mechanistic plausibility of our findings. The use of machine learning and assessment of net clinical benefit also enhanced the potential clinical utility of our diagnostic model. Nevertheless, several limitations should be acknowledged. First, the roles of some identified proteins, such as P-gp and CS, in PB-induced AKI remain insufficiently understood and require further experimental validation. Second, although UPLC-HRMS provides high-throughput metabolic profiling, the technique primarily captures high-abundance metabolites and may overlook low-abundance metabolites of potential biological importance. Future studies using targeted metabolomics are warranted to address this gap. Third, the mechanistic conclusions are derived primarily from a rat model of PB-induced AKI, while the human component consists of only a small cohort; therefore, direct extrapolation from rodents to the human kidney is limited. Fourth, AKI diagnosis relied solely on serum creatinine because urine output data were not systematically recorded. Consequently, non-AKI may have been misclassified as non-AKI, biasing the metabolomic classifier toward the null and possibly reducing performance. The reported AUC of 1.000 may be optimistic; future prospective studies should use both criteria. Fifth, although adequate for initial model development, the limited sample size and single-center design may constrain the model’s applicability to diverse patient populations. Multicenter prospective studies are needed to further validate the robustness and utility of the model.

## 4. Methods and Materials

### 4.1. Chemicals

PB1 (Lot No. CFW-VN977800U) and ILE-PB1 (Lot No. CFW-VN978200U) were purchased from CFW Laboratories Inc. (Wilmington, DE, USA), while PB2 (Lot No. 2336803P-YR-01), PB3 (Lot No. 2336804P-YR-01), and PB6 (Lot No. 2336805P-YR-01) were obtained from Standard Pharmaceuticals (Guangzhou, China) Co., Ltd. (Shanghai, China). Polymyxin B sulfate (for injection) was purchased from Shanghai First Biochemical Pharmaceutical Co., Ltd. (Shanghai, China). Formic acid and ammonium formate were purchased from Sigma-Aldrich (St. Louis, MO, USA), chromatographic-grade methanol and acetonitrile from Fisher Scientific (Waltham, MA, USA), and ultrapure water was prepared via a Milli-Q water purification system (Merck Millipore, Billerica, MA, USA). All other reagents were of analytical grade.

### 4.2. Study Design and Patients

This retrospective observational cohort study enrolled hospitalized adult patients who received intravenous polymyxin B (PB) for confirmed or suspected bacterial infections between April 2023 and September 2025 at China-Japan Friendship Hospital. Ethical approval was obtained from the Ethics Committee of China-Japan Friendship Hospital (approval no. 2023-KY-213-1), and written informed consent was obtained from each patient. This study was conducted in accordance with the Declaration of Helsinki.

The inclusion criteria were as follows: (a) age ≥ 18 years; (b) intravenous PB treatment for more than 4 days; and (c) TDM. The exclusion criteria were as follows: (a) receipt of renal replacement therapy or extracorporeal membrane oxygenation; (b) death within 48 h after the initiation of PB treatment; and (c) absence of a baseline serum creatinine value. Patients were divided into AKI and non-AKI groups. AKI was defined according to the Kidney Disease: Improving Global Outcomes (KDIGO) criteria as an increase in serum creatinine of ≥0.3 mg/dL (>26.5 μmol/L) within 48 h or a ≥1.5-fold increase from baseline [48]. Blood samples for TDM were centrifuged at 1500× *g* for 5 min, and the plasma was stored at −80 °C. TDM points: C_min_, trough concentration immediately before the next dose at steady state; C_max_, peak concentration 2 h post-infusion at steady state; C_total_, total PB1 + ILE-PB1 + PB2 + PB3 + PB6 combined concentration from each TDM sample; AUC_ss,24h_, estimated using a one-compartment model fit to each patient’s available sampling points.

### 4.3. Animal Experiments

Forty-eight 180g male Sprague–Dawley rats (Sibeifu Biotechnology Co., Ltd., Beijing, China) were housed four per cage with free access to food and water. Ethical approval was obtained from the Institutional Animal Care and Use Committee of China-Japan Friendship Hospital (Approval No. 00004110). All procedures were performed in accordance with the Animal Research: Reporting of In Vivo Experiments guidelines and the National Institutes of Health Guide for the Care and Use of Laboratory Animals. The rats were randomly assigned to a control group or one of three PB groups. The control animals (*n* = 12) received normal saline by subcutaneous injection twice daily for 10 days. Animals in the PB groups (*n* = 12 per group) received polymyxin B sulfate at doses of 3, 6, or 12 mg·kg^−1^·per injection, administered subcutaneously twice daily for 10 days. Twelve hours after the final injection, the rats were euthanized with carbon dioxide, and blood, urine, and kidney samples were collected and stored at −80 °C.

### 4.4. Detection of PB Components in Plasma and Kidneys

For sample preparation, 150 μL of plasma was mixed with an equal volume of 5% (*w*/*v*) trichloroacetic acid (TCA), vortexed for 5 min, and centrifuged at 15,000× *g* at 4 °C for 10 min. A 100 μL aliquot of the supernatant was then diluted with 100 μL of water, vortexed for 60 s, and transferred to a vial for analysis. Kidney tissue samples were homogenized in saline (1:3, *w*/*v*) and centrifuged at 15,000× *g* at 4 °C for 10 min; the supernatant was processed in the same manner as the plasma samples.

Chromatography: instrument, Ultimate 3000 LC system (Thermo Fisher Scientific, Waltham, MA, USA); column, Acquity Peptide CSH-C18 (2.1 × 100 mm, 1.7 μm; Waters, Milford, MA, USA), maintained at 40 °C; mobile phase, (A) water containing 1% formic acid and (B) methanol/acetonitrile (1:1, *v*/*v*) containing 1% formic acid; gradient elution at 0.2 mL/min: 0–2 min, 25% B; 2–4.5 min, 25–30% B; 4.5–5.5 min, 30–95% B; 5.5–6.5 min, 95% B; 6.5–6.7 min, 95–25% B; 6.7–9 min, 25% B; injection volume, 5 μL; autosampler temperature, 4 °C.

MS conditions: instrument, Q Exactive Orbitrap mass spectrometer equipped with an electrospray ionization (ESI) source operated in positive-ion mode (Thermo Fisher Scientific, Waltham, MA, USA); spray voltage, 2500 V; vaporizer temperature, 400 °C; capillary temperature, 320 °C; S-lens radio frequency (RF), 50 V; full MS resolution, 70,000; dd-MS^2^ resolution, 17,500; automatic gain control (AGC) target, 2 × 10^5^; maximum injection time (IT), 100 ms; normalized collision energy (NCE), 22 eV. Monitored ions: *m*/*z* 595.3926 [M + H]^+^ (PB2, PB3), 602.4004 [M + H]^+^ (PB1, ILE-PB1), and 610.3979 [M + H]^+^ (PB6).

The method validation results for the individual components of PB, including linear ranges, calibration ranges, internal standards, matrix effects, extraction recovery, accuracy, and other relevant parameters, are provided in Supplementary File S2.

### 4.5. Untargeted Metabolomics

For sample preparation, a 50 μL aliquot was mixed with 450 μL of methanol-acetonitrile (1:1, *v*/*v*) containing internal standards (ISs; propranolol and tolbutamide, 200 ng/mL each), vortexed for 1 min, and centrifuged at 15,000× *g* at 4 °C for 10 min. A 200 μL aliquot of the supernatant was then stored at 4 °C until LC-MS analysis. Kidney tissue samples were homogenized in ultrapure water containing 50% methanol (1:10, *w*/*v*) and centrifuged at 15,000× *g* at 4 °C for 10 min; a 50 μL aliquot of the supernatant was processed in the same manner as the plasma samples.

Chromatographic conditions: instrument, Ultimate 3000 LC system (Thermo Fisher Scientific, Waltham, MA, USA); column, ACQUITY BEH C18 (1.7 μm, 2.1 × 50 mm; Waters, Milford, MA, USA), maintained at 30 °C; mobile phase, (A) water containing 0.1% formic acid and 2.5 mM ammonium formate and (B) acetonitrile; gradient elution at 0.25 mL/min: 0–1 min, 95% A; 1–5 min, 95–40% A; 5–8 min, 40–0% A; 8–11 min, 0% A; 11–14 min, 0–40% A; 14–15 min, 40–95% A; 15–18 min, 95% A; injection volume, 5 μL; autosampler temperature, 4 °C.

MS conditions: instrument, Q Exactive Orbitrap mass spectrometer equipped with an electrospray ionization (ESI) source operated in both positive (+3 kV) and negative (−3 kV) ion modes (Thermo Fisher Scientific, Waltham, MA, USA); vaporizer temperature, 350 °C; capillary temperature, 320 °C; S-lens radio frequency (RF), 50 V. Full MS: resolution, 70,000; *m*/*z* range, 70–1000. dd-MS^2^: resolution, 17,500; automatic gain control (AGC) target, 1 × 10^5^; maximum injection time (IT), 50 ms; normalized collision energy (NCE), 20, 40, and 60 eV.

Untargeted metabolomics analysis was performed on the 125 plasma samples. The pooled serum QC samples were evenly distributed throughout the sample analysis process to monitor the system stability, with an injection frequency of 1/10 (one QC sample was analyzed after 10 analytical samples).

### 4.6. Metabolic Pathway Analysis

Data processing and endogenous metabolite annotation were performed using an in-house database [49,50]. Missing values were imputed by the half-minimum method (features with >50% missingness were excluded). Data were normalized by probabilistic quotient normalization (PQN) using QC as reference, followed by log2 transformation. MetaboAnalyst 6.0 (https://www.metaboanalyst.ca/, accessed on 5 September 2026) was used for multivariate statistical analyses, including principal component analysis (PCA) and partial least squares discriminant analysis (PLS-DA). Differentially abundant metabolites were identified using a variable importance in projection (VIP) score > 1.0. Pathways with an impact value > 0.1, as identified by the “MS Peaks to Pathways” module of MetaboAnalyst 6.0, were selected for further analysis.

### 4.7. MSI Analyses

MSI analysis of PB and creatine in kidney sections was performed using an airflow-assisted desorption electrospray ionization (AFADESI) ambient ion source (Beijing Victor Technology Co., Ltd., Beijing, China) coupled to a Q-OT-qIT hybrid mass spectrometer (Orbitrap Exploris 240; Thermo Fisher Scientific, Waltham, MA, USA). The following parameters were used: spray voltage, ±4.5 kV; spray gas pressure, 0.6 MPa; extraction gas flow, 45 L/min; *X*-axis scanning speed, 0.2 mm/s; *Y*-axis step size, 0.2 mm; mass range, 70–1000 Da.

For creatinine imaging, an iMScope QT system (Shimadzu, Kyoto, Japan) equipped with an integrated optical microscope, a matrix-assisted laser desorption/ionization (MALDI) source, and a quadrupole time-of-flight (Q-TOF) analyzer was used. The scanning area was determined by optical microscopy, and the sections were irradiated with a 355 nm YAG laser under the following conditions: 200 laser shots at 2000 Hz; 10 μm spot diameter; 60% laser intensity; detector voltage, 2.7 kV; pitch, 50 μm (spatial resolution); mass range, 70–500 Da.

### 4.8. Reverse Transcription Quantitative Real-Time PCR (RT-qPCR) Analysis

RT-qPCR was performed using an ABI 7300 Real-Time PCR System (Applied Biosystems, Foster City, CA, USA) and ChamQ SYBR Color qPCR Master Mix (2×) (Vazyme Biotech Co., Ltd., Nanjing, China). The thermal cycling protocol was as follows: initial denaturation at 95 °C for 3 min, followed by 40 cycles of denaturation at 95 °C for 5 s and annealing at 55 °C for 30 s (primer sequences are listed in Table S1). Primer efficiencies of 95–105% were determined by standard curve analysis; 6 rats were sampled per group with technical triplicates for each sample. Normalization was performed by the 2^−ΔΔCt^ method, and statistical analysis was performed in GraphPad Prism 9.0 using Shapiro–Wilk and Levene’s tests for assumption checking, followed by one-way ANOVA with Tukey’s test or Kruskal–Wallis with Dunn’s correction as appropriate.

### 4.9. Molecular Docking

Crystal structures of human P-glycoprotein (P-gp; PDB ID: 6QEX; resolution: 3.60 Å) and its rat ortholog (PDB ID: 4M2S; resolution: 4.40 Å) were retrieved from the RCSB Protein Data Bank (RCSB PDB; www.rcsb.org). The structures of PB, human P-gp, and rat P-gp were converted to PDBQT format using AutoDockTools 1.5.6, and the conformations with the highest docking scores were visualized using PyMOL 1.7.6. A binding score < −5.0 kcal/mol was considered indicative of satisfactory binding.

### 4.10. Biochemical and Histopathological Analyses 

Blood biochemical parameters, including serum creatinine, blood urea nitrogen (BUN), uric acid (UA), glucose (GLU), total cholesterol (TC), and triglycerides (TG), were measured using an automated biochemical analyzer (Hitachi High-Technologies Corporation, Tokyo, Japan). Hematological parameters were assessed using a hematology analyzer (Sysmex XN-1000VB1, Tokyo, Japan), and urinalysis was performed using a urine analyzer (URIT UC280, Guilin, China). Rat kidney tissues were fixed in 4% paraformaldehyde, embedded in paraffin, stained with hematoxylin and eosin (H&E), and imaged using an automated digital slide-scanning system (Roche Diagnostics, Mannheim, Germany) for morphological evaluation of the glomeruli and tubules. For histopathological evaluation, one kidney per rat was used. Renal tissue was fixed, embedded, sectioned, and stained with HE. For this purpose, the assessor was blinded to treatment assignment. Scoring items included tubular injury, interstitial inflammation, and glomerular injury. The semiquantitative 0–4 scale was defined as 0, none; 1, <10%; 2, 11–25%; 3, 26–45%; and 4, >46%. When multiple items were scored, the total score was calculated, and the animal served as the final unit of analysis. From each section, five non-overlapping fields were randomly selected at 400× high-power magnification; when both the cortex and medulla were covered, fields were selected proportionally. The mean field score was used as the animal’s score. For multiple sections, scores were first averaged within each section. Sections and fields were not treated as independent observations; n referred to the number of animals.

### 4.11. Statistical Analysis

The PB TDM model was developed and evaluated by logistic regression using SPSS Statistics 23.0 (IBM Corp., Armonk, NY, USA), and its diagnostic performance was assessed by receiver operating characteristic (ROC) curve analysis. Intergroup differences were evaluated by one-way analysis of variance, followed by Tukey’s post hoc test for multiple comparisons. Normality and homogeneity of variance were assessed using the Shapiro–Wilk and Levene’s tests, respectively. A *p* value < 0.05 was considered statistically significant.

### 4.12. Machine Learning

Using the CNSknowall platform (http://cnsknowall.com, accessed on 20 September 2026), 10 features were selected from the differentially abundant metabolites identified in patients (VIP > 1.0, *p* < 0.05) with the R package xgboost, and decision curve analysis (DCA) was performed using the R package ggDCA. A 6-metabolite diagnostic classifier for the diagnosis of PB-associated AKI was constructed using a machine learning algorithm on the OECloud platform (https://cloud.oebiotech.com, accessed on 20 September 2026). The participants were randomly stratified into a training dataset *(n* = 87) and a test set (*n* = 38). We first developed a series of machine learning models, including random forest, logistic regression, eXtreme Gradient Boosting (XGBoost), and least absolute shrinkage and selection operator (LASSO), using the training dataset with 2000 stratified bootstrap replicates. XGBoost was selected as the best-performing model according to its accuracy, precision, sensitivity, specificity, and area under the receiver operating characteristic curve (AUC). SHapley Additive exPlanations (SHAP) values were calculated and plotted to interpret the contributions of individual features to the model’s predictions and provide insight into the relative importance of each predictor in assessing AKI risk.

## 5. Patents

Application of metabolic markers in preparing products for monitoring PB nephrotoxicity and related products. Xiaoxue Wang; Lihong Liu; Pengmei Li; Ying Liu; Wei Qin; Wenqian Chen; Bo Li; Shu Li. Chinese Patent Authorization Number: CN 118777488 B.

## Figures and Tables

**Table 1:** Baseline Characteristics and In-Hospital Mortality by AKI Status and Concentration Group (C_min_ vs. C_max_).

Characteristic	Non-AKI (*n* = 60)	AKI (*n* = 23)	*p* Value
Age, years	67.0 [57.0, 74.0]	69.0 [64.5, 77.5]	0.231
Female, %	23.3	13.0	0.368
Male, %	76.7	87.0	—
BMI, kg/m^2^	22.0 [19.0, 26.7]	21.8 [19.6, 23.5]	0.630
Alive, *n* (%)	33 (55.0)	9 (39.1)	—
Dead, *n* (%)	27 (45.0)	14 (60.9)	—
Mortality, %	45.0	60.9	0.186
C_max_, *n* (%)	73 (56.2)	28 (50)	—
C_min_, *n* (%)	57 (43.8)	28 (50)	—

## Data Availability

The datasets that support the findings of this study are not publicly available due to ethical and privacy restrictions and are available from the corresponding author upon reasonable request.

## Supplementary Materials

The following supporting information can be downloaded at https://www.mdpi.com/article/10.3390/antibiotics15100966/s1.

## Abbreviations

α-KG	α-ketoglutarate
ACs	Acylcarnitines
ACS	Acyl-CoA synthetase
AFADESI-MSI	Airflow-assisted desorption electrospray ionization-mass spectrometry imaging
AKI	Acute kidney injury
AUC	Area under the curve
AUC_ss,24h_	24-h area under the concentration-time curve at steady state
BCAAs	Branched-chain amino acids
BUN	Blood urea nitrogen
CD36	Cluster of differentiation 36
C_max_	Peak concentration
C_min_	Trough concentration
CPT1	Carnitine palmitoyltransferase 1
CPT2	Carnitine palmitoyltransferase 2
CS	Citrate synthase
DHA	Docosahexaenoic acid
DPA	Docosapentaenoic acid
FA	Fatty acid
FAO	Fatty acid oxidation
FABP3	Fatty acid-binding protein 3
IDH	Isocitrate dehydrogenase
ILE-PB1	Isoleucine-polymyxin B1
KDIGO	Kidney Disease: Improving Global Outcomes
KEGG	Kyoto Encyclopedia of Genes and Genomes
LPCs	Lysophosphatidylcholines
LPEs	Lysophosphatidylethanolamines
MALDI	Matrix-assisted laser desorption/ionization
MDH	Malate dehydrogenase
OAs	Organic acids
PCA	Principal component analysis
PC	Phosphatidylcholine
PCs	Phosphatidylcholines
PDB	Protein data bank
PDH	Pyruvate dehydrogenase
P-gp	P-glycoprotein
PLS-DA	Partial least squares discriminant analysis
PPARα	Peroxisome proliferator-activated receptor-α
PB	Polymyxin B
PB1	Polymyxin B1
PB2	Polymyxin B2
PB3	Polymyxin B3
PB6	Polymyxin B6
ROC	Receiver operating characteristic
ROS	Reactive oxygen species
SHAP	SHapley Additive exPlanations
SMs	Sphingomyelins
TCA	Tricarboxylic acid
TDM	Therapeutic drug monitoring
TICs	Total ion chromatograms
XGBoost	eXtreme Gradient Boosting
