# Supplementary material for: Diagnostic Biomarkers and Metabolic Mechanisms of Polymyxin B-Induced Acute Kidney Injury
Source: Antibiotics (Basel). 2026 Sep 30;15(10):966. doi: 10.3390/antibiotics15100966 (PMC13700662; doi:10.3390/antibiotics15100966)
Supplement: Supplementary file 1 [file antibiotics-15-00966-s001.zip › Supplementary File S1.pdf]

Supplementary Information

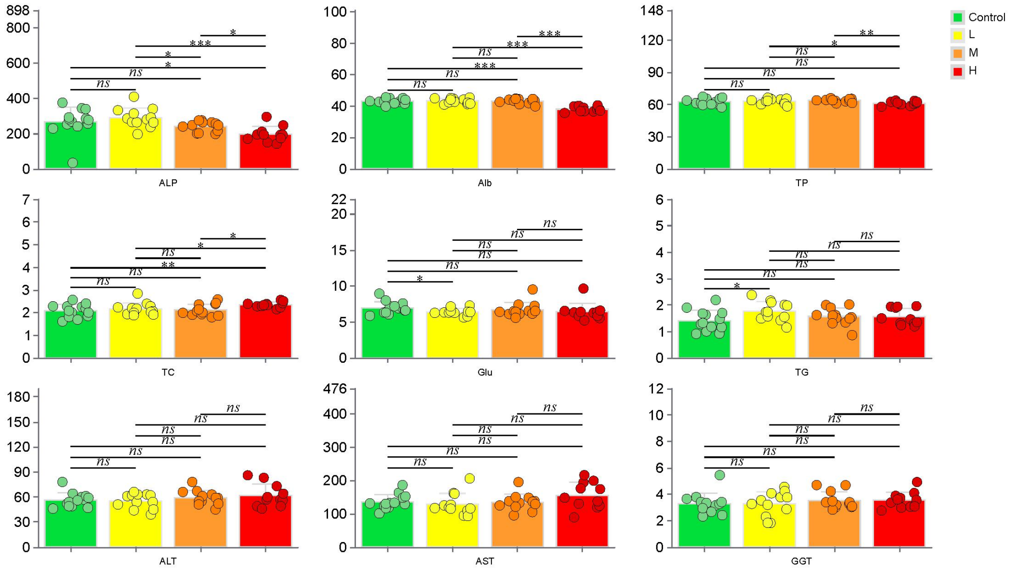

Figure S1. Bar plots showing changes in blood biochemical parameters, including alkaline phosphatase (ALP), albumin (Alb), total protein (TP), glucose (Glu), total cholesterol (TC), triglyceride (TG), alanine aminotransferase (ALT), aspartate aminotransferase (AST), and gamma-glutamyl transferase (GGT) on day 10 after polymyxin B (PB) administration at low (L, 3 mg/kg), medium (M, 6 mg/kg), or high (H, 12 mg/kg) doses. Data are represented as mean± SD (*n* = 12 per group). Significant differences were determined by Student's t-test (*, *P* < 0.05; **, *P* < 0.01; ***, *P* < 0.001; ns, not significant).

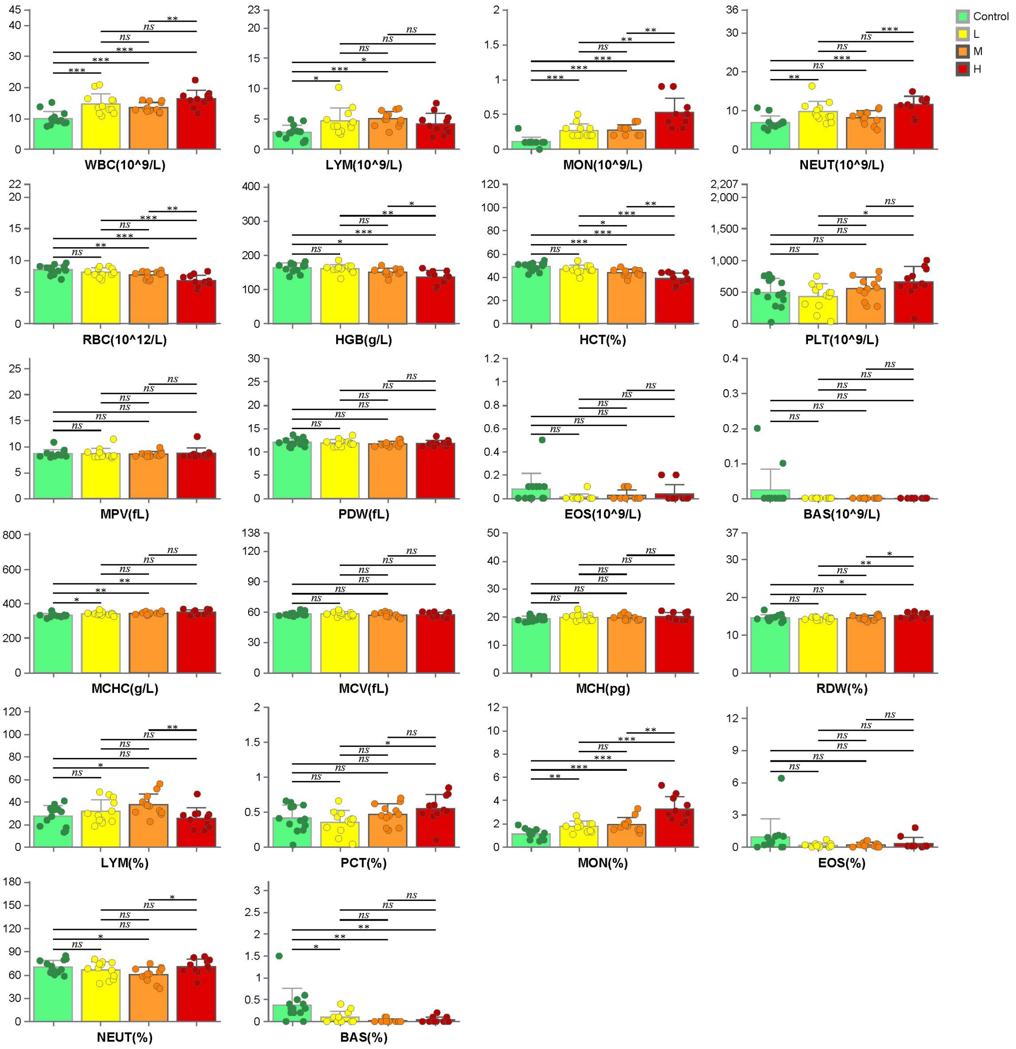

Figure S2. Bar plots showing changes in hematological parameters, including white blood cell (WBC), lymphocyte (LYM), monocyte (MON), neutrophil (NEUT), red blood cell (RBC), hemoglobin (HGB), hematocrit (HCT), platelet (PLT), mean platelet volume (MPV), platelet distribution width (PDW), eosinophil (EOS), basophil (BAS), mean corpuscular hemoglobin concentration (MCHC), mean corpuscular volume (MCV), mean corpuscular hemoglobin (MCH), and red cell distribution width (RDW) on day 10 after polymyxin B (PB) administration at low (L, 3 mg/kg), medium (M, 6 mg/kg), or high (H, 12 mg/kg) doses. Data are represented as mean ± SD (n = 12 per group). Significant differences were determined by Student's t-test (*, $P < 0.05$; **, $P < 0.01$; ***, $P < 0.001$; ns, not significant).

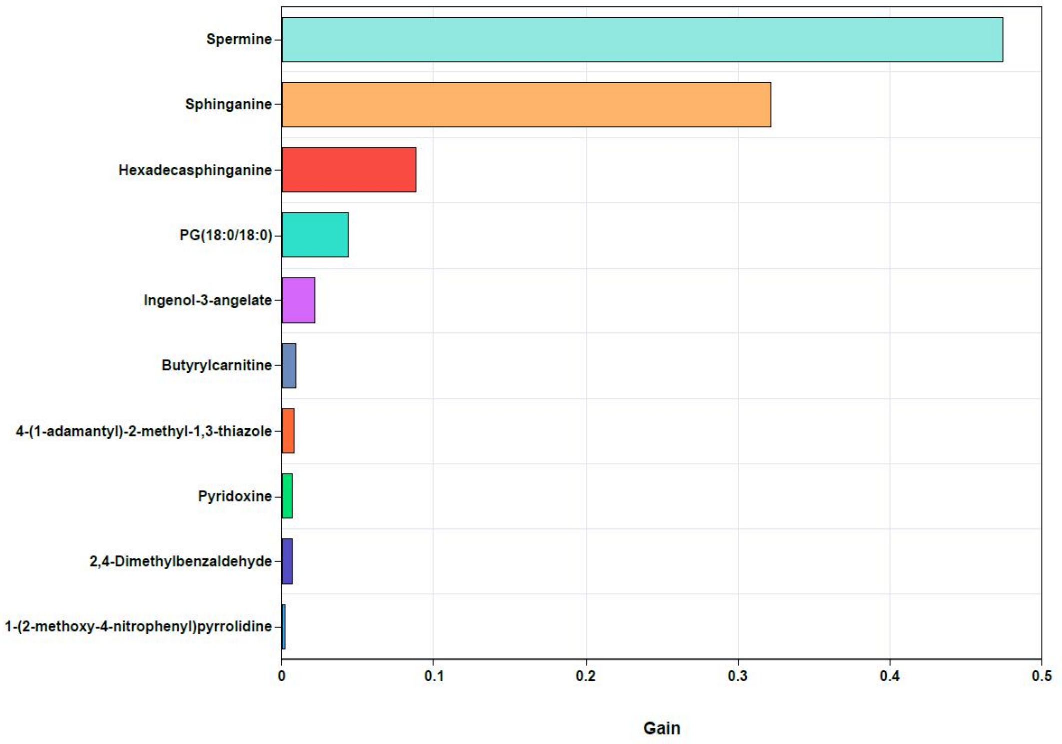

Figure S3. Feature screening chart using eXtreme Gradient Boosting **(**XGBoost).

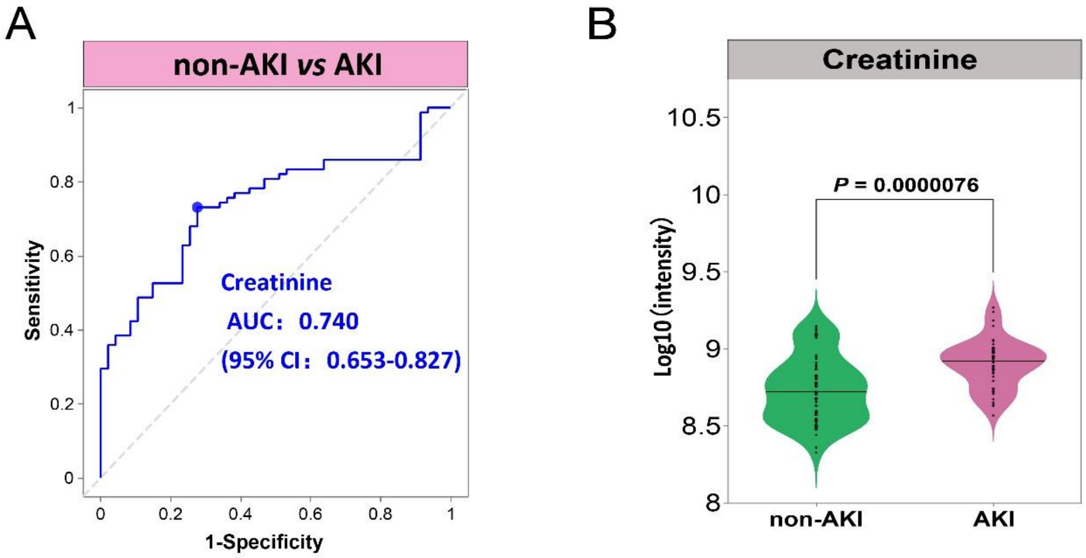

Figure S4. Receiver operating characteristic (ROC) curve (A) and log10-transformed relative abundance (B) of creatinine. Statistical significance was determined by two-sided Wilcoxon rank-sum test.

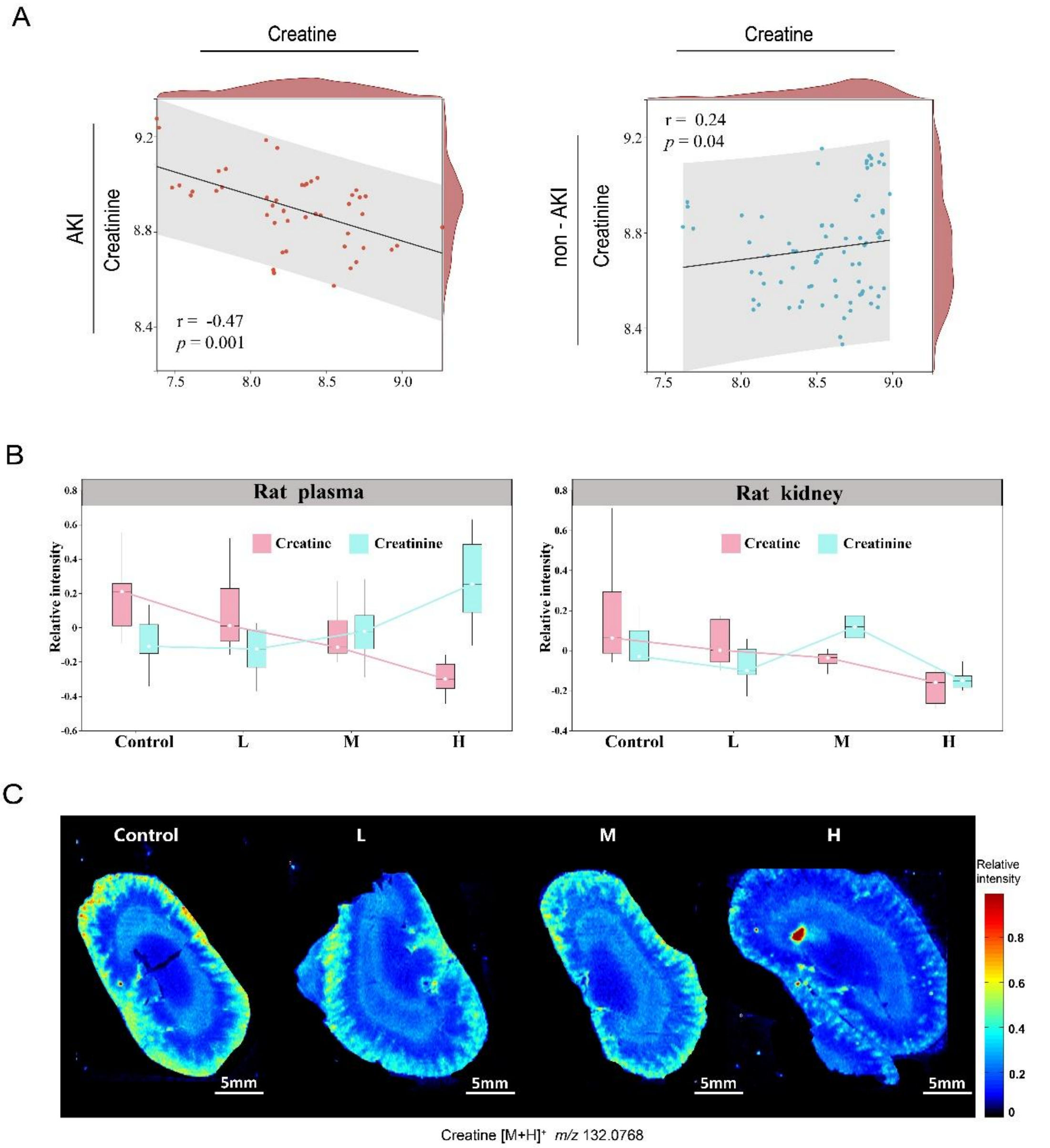

Figure S5. (A) Spearman correlation analysis between creatinine (a key clinical indicator of renal function) and creatine in non-AKI and PB-induced AKI groups separately. (B) Boxplots of creatine and creatinine relative abundance in rat plasma and kidney from control rats and rats administered low (L, 3 mg/kg), medium (M, 6 mg/kg), or high (H, 12 mg/kg) doses of polymyxin B (PB). Statistical significance was determined by Kruskal-Wallis test with Dunn's post-hoc analysis. The center line represents the median, the box bounds indicate the interquartile range (IQR), and the whiskers span 1.5-fold the IQR. (C) Air flow-assisted desorption electrospray ionization mass spectrometry (AFADESI-MS) images showing

creatine distribution in kidney tissue after PB exposure.

Table S1. The primer sequences for genes

No.	Gene Name	Primer Name	Primer Sequence (5'—> 3')
1	fatty acid-binding protein 3 (*Fabp3*)	Fabp3F	ACCTGGAAGCTAGTGGACAG
		Fabp3R	TGATGGTAGTAGGCTTGGTCAT
2	acyl-CoA synthetase (*Acs*),	AcsF	TCCTGACGTTTGGAACGGC
		AcsR	CTCCCTCAATCCCCACAGAC
3	citrate synthase (*Cs*)	CsF	GGACAATTTTCCAACCAATCTGC
		CsR	TCGGTTCATTCCCTCTGCATA
4	isocitrate dehydrogenase (*Idh*)	IdhF	GTGGAGATGGACGGTGACGAGAT
		IdhR	TGGTCTGGTCACGGTTTGGAA
5	pyruvate dehydrogenase beta (*Pdhb*)	PdhbF	AGGAGGGAATTGAATGTGAGGT
		PdhbR	ACTGGCTTCTATGGCTTCGAT
6	malate dehydrogenase (*Mdh*)	MdhF	ATTGCCTCAAAGGTTGTGATGTG
		MdhR	GGATGGTGGAGTTCACTGGGTT
7	cluster of differentiation 36 (*Cd36*)	Cd36F	CCTTAAAGGAATCCCCGTGT
		Cd36R	TGCATTTGCCAATGTCTAGC
8	carnitine palmitoyltransferase 2 (*Cpt2*)	Cpt2F	CTATCCCTAAACTTGAAGACACCA
		Cpt2R	TCCCAATGCCGTTCTCAAAAT
9	peroxisome proliferator-activated receptor α (*Ppara*)	PPARα-F	AGAGGTCCGATTCTTCCA
		PPARα-R	AGCATCCCGTCTTTGTTC
10	carnitine palmitoyltransferase 1 (*Cpt1*)	Cpt1-F	GGCGGTACTTCAAGGTCTG
		Cpt1-R	GCTGTGGCTCTGAGGGATC
11	uncoupling protein 3 (*Ucp3*)	Ucp3F	CTGCACCGCCAGATGAGTTT
		Ucp3R	ATCATGGCTTGAAATCGGACC
12	acyl-CoA thioesterase 2 (*Acot2*)	Acot2F	GTTGTGCCAACAGGATTGGAA
		Acot2R	GCTCAGCGTCGCATTTGTC
13	lactate dehydrogenase (*Ldh*)	LdhF	TGTCTCCAGCAAAGACTACTGT
		LdhR	GACTGTACTTGACAATGTTGGGA
14	succinate dehydrogenase (*Sdh*)	SdhF	GGAACACTCCAAAAACAGACCT
		SdhR	CCACCACTGGGTATTGAGTAGAA
15	Mouse actin betaNM_007393(Reference Gene)	actin-F1566	GTCCCTCACCCTCCCAAAAG
		actin-R1831	GCTGCCTCAACACCTCAACCC

Table S2. Predicted Toxicity Endpoints of Polymyxin B

Toxicity Endpoint / Compound	CYP450 Metabolism	CYP450 Inhibition	P-gp Substrate	P-gp Inhibitor	Target Organs/Tissues	LD_{50} Value
PB1	None	None	Yes	No	Kidney, Liver, Blood, Gastrointestinal System, Cardiovascular System	Rat：56 mg/kg; Mouse：38 mg/kg
PB2	None	None	Yes	No	Lung, Kidney, Liver, Blood, Gastrointestinal System, Cardiovascular System	Rat：34 mg/kg; Mouse：24 mg/kg
PB3	None	None	Yes	No	Kidney, Liver, Blood, Gastrointestinal System, Cardiovascular System	Rat：56 mg/kg; Mouse：38 mg/kg
ILE-PB1	None	None	Yes	No	Lung, Kidney, Liver, Blood, Gastrointestinal System, Cardiovascular System	Rat：41 mg/kg; Mouse：23 mg/kg

Table S3. P-glycoprotein (P-gp) Binding Site of PB

Species	PB1	PB2
**Human**	Leu-65, Met-69, Met-192, Ala-229, Trp-232,Phe-303,Ile-306, Phe-336, Ile-340, Phe-343, Phe-728, Phe-732, Ala-871, Met-949, and Phe-983	Leu-65, Ala-229, Leu-236, Ile-299, Trp-232, Phe-303, Ile-306, Phe-335, Phe-336, Leu-339, Ile-340, Phe-343, Leu-724, Phe-728, Phe-770, Met-876, Met-949, Phe-983, and Phe-994
**Rat**	Leu-64, Phe-299, Leu-335, Ile-336, Phe-339, Phe-724, Met-945, Ala-983, and Val-987	Leu-64, Met-188, Leu-335, Ile-336, Phe-339, Ile-864, Ala-865, Met-872, Met-945, Phe-979, and Met-982

Table S4. Effect of different doses of PB on body weight and organ-to-body weight ratio in rats (n=12 per group, Mean±SD)

Group	Body weight(g)	Kidney	
		Weight (g)	weight index (%)
Control (0 mg/kg)	272.69±15.32	1.97±0.23	0. 72±0. 06
L (3 mg/kg)	266.08±13.24	1.94±0.13	0. 73±0. 04
M (6 mg/kg)	256.92±16.13**	1.83±0.20	0. 72±0. 04
H (12 mg/kg)	247.18±16.45***	1.86±0.22	0. 75±0. 05

P*<0.01,*P*<0.001 vs. the control group.

Table S5. Effects of Polymyxin B on Blood Biochemical Parameters in Rats (n=12 per group, Mean±SD)

Time	Parameter	0 mg/kg	3 mg/kg	6 mg/kg	12 mg/kg
Day5	Urea **(4.0-9.3 mmol/L)**	4.34±0.69	4.46±0.49	4.63±1.04	6.03±0.56***
	Creatinine **(31-48 μmol/L)**	50.82±8.55	50.91±8.84	42.20±10.09	41.89±7.82
Day8	Urea **(4.0-9.3 mmol/L)**	4.36±0.52	4.79±0.59	6.45±1.03***	8.45±2.09***
	Creatinine **(31-48 μmol/L)**	53.00±4.37	47.58±1.71	53.00±3.46	59.91±6.04**
Day10	Urea **(4.0-9.3 mmol/L)**	4.68±1.03	4.43±1.91	4.85±1.64	8.30±2.66***
	Creatinine **(31-48 μmol/L)**	30.62±2.44	27.33±2.56	28.42±2.98	33.45±4.05 (*P*=0.05)
	Alanine Aminotransferase (ALT) **(23-67 U/L)**	56.23±8.30	55.33±8.55	59.25±8.34	61.82±12.88
	Aspartate Aminotransferase (AST) **(61-153 U/L)**	135.77±21.09	128.58±30.99	134.42±23.88	155.09±37.65

Gamma-Glutamyl Transferase (GGT) **(0-8 U/L)**	3.27±0.77	3.27±0.87	3.52±0.62	3.55±0.58
Alkaline Phosphatase (ALP) **(210-280 U/L)**	266.32±78.79	288.92±52.09	239.58±27.85	195.82±41.32*
Blood Glucose (Glu) **(5.1-9.2 mmol/L)**	6.94±0.81	6.37±0.45	6.69±0.97	6.40±1.10
Total Protein (TP) **(40-80 g/L)**	62.58±2.91	63.15±2.38	63.75±1.41	61.12±1.78
Albumin (Alb) **(27-33 g/L)**	43.12±1.54	43.23±1.55	43.13±1.50	37.94±1.57***
Total Cholesterol (TC) **(0.7-2.5 mmol/L)**	2.08±0.27	2.16±0.26	2.12±0.25	2.35±0.11
Triglyceride (TG) **(0.4-2.1 mmol/L)**	1.41±0.38	1.77±0.34*	1.57±0.31	1.55±0.30

Compared with the 0 mg/kg group, **P*<0.05, ***P*<0.01, ****P*<0.001.

Table S6. Effects of Polymyxin B on Urinary Parameters in Rats (n=12 per group, Mean±SD)

Time	Parameter	0 mg/kg	3 mg/kg	6 mg/kg	12 mg/kg
	Protein (PRO) **(<0.15 g/L)**	0	0	0.01±0.04	0.01±0.04
	Occult Blood **(<10 CELL/μL)**	58.85±68.73	22.08±27.57	21.67±27.26	46.36±31.56
	Glucose (GLU) **(<2.8 mmol/L)**	0	0	0	0
**Day5**	White Blood Cell (WBC) **(0 CELL/μL)**	0	0	0	0
	Ketones **(0 mmol/L)**	0	0	0	0
	Nitrite **(0 μmol/L)**	Neg	Neg	Neg	Neg
	Urobilinogen	Normal	Normal	Normal	Normal

	**(3.2-16 μmol/L)**				
	Bilirubin **(0 μmol/L)**	0	0	0	0
	Specific Gravity (SG) **(1.010-1.025)**	1.012±0.003	1.011±0.004	1.012±0.004	1.018±0.008*
	pH **(5.5-7.0)**	6.96±0.63	7.00±0.20	6.92±0.40	6.23±0.49**
	Vitamin C **(0 mmol/L)**	0	0	0	0.05±0.17
**Day8**	Protein **(<0.15 g/L)**	0.01±0.04	0.16±0.04***	0.45±0.40**	0.60±0.44***
	Occult Blood **(<10 CELL/μL)**	18.08±27.28	4.17±4.93	20.83±20.70	25.00±27.30
	Glucose(GLU) **(<2.8 mmol/L)**	0	0	1.84±2.34*	2.51±2.48**
	White Blood Cell (WBC) **(0 CELL/μL)**	0	0	5.00±7.07*	9.09±20.09
	Ketones **(0 mmol/L)**	0	0	0	0
	Nitrite **(0 μmol/L)**	Neg	Neg	Neg	Neg
	Urobilinogen **(3.2-16 μmol/L)**	Normal	Normal	Normal	Normal
	Bilirubin **(0 μmol/L)**	0	0	0	0
	Specific Gravity (SG) **(1.010-1.025)**	1.013±0.005	1.016±0.002	1.019±0.004**	1.025±0.005***
	pH **(5.5-7.0)**	7.08±0.43	7.75±0.75*	6.83±0.42	5.91±0.51***
	Vitamin C **(0 mmol/L)**	0.19±0.44	0.05±0.17	0.52±1.54	0.42±0.80
**Day10**	Protein **(<0.15 g/L)**	0.05±0.09	0.14±0.10	0.20±0.26	0.50±0.39***
	Occult Blood **(<10 CELL/μL)**	6.92±8.89	12.08±21.74	4.58±7.49	9.55±10.33
	Glucose (GLU)	0	0	0.23±0.77	2.54±4.03*

**(<2.8 mmol/L)**				
White Blood Cell (WBC) **(0 CELL/μL)**	0	1.25±4.15	2.50±5.59	11.82±19.69*
Ketones **(0 mmol/L)**	0	0	0.08±0.19	0.09±0.19
Nitrite **(0 μmol/L)**	Neg	Neg	Neg	Neg
Urobilinogen **(3.2-16 μmol/L)**	Normal	Normal	Normal	Normal
Bilirubin **(0 μmol/L)**	0	0	0	0
Specific Gravity (SG) **(1.010-1.025)**	1.013±0.004	1.013±0.004	1.018±0.006*	1.022±0.005***
pH **(5.5-7.0)**	7.19±0.50	7.92±0.67**	6.79±0.52	6.27±0.75**
Vitamin C **(0 mmol/L)**	0.05±0.16	0.22±0.42	0.85±1.52	0.11±0.23

Compared with the 0 mg/kg group, *P<0.05, **P<0.01, ***P<0.001.

Table S7. Effects of Polymyxin B on Hematological Parameters in Rats (n=12 per group, Mean±SD)

**Parameter**	**0 mg/kg**	**3 mg/kg**	**6 mg/kg**	**12 mg/kg**
White blood cell (WBC) **(5.0-15.0 10^9/L)**	9.92±2.18	14.64±3.14***	13.52±1.51***	16.29±2.60***
Lymphocyte (LYM) **(4.0-10.0 10^9/L)**	2.77±1.17	4.67±2.06*	5.03±1.16***	4.21±1.67*
Monocyte (MON) **(0.2-1.7 10^9/L)**	0.11±0.06	0.27±0.09***	0.28±0.07***	0.53±0.20***
Neutrophil (NEUT) **(1.1-4.0 10^9/L)**	6.94±1.60	9.70±2.54**	8.13±1.71	11.52±2.06***
Red blood cell (RBC) **(5.0-12.0 10^12/L)**	8.50±0.71	8.10±0.65	7.73±0.53**	6.79±0.83***
Hemoglobin (HGB) **(111-180 g/L)**	163.54±13.01	160.00±12.11	150.75±10.04*	136.27±17.75***

Hematocrit (HCT) **(36.0-59.0 %)**	49.13±3.40	46.79±3.38	43.73±2.88***	38.78±4.52***
Platelet (PLT) **(140-600 10^9/L)**	530.42±174.38	463.18±165.71	552.83±176.03	718.80±152.54*

Compared with the 0 mg/kg group， *P<0.05, **P<0.01,***P<0.001.

Table S8. Partial least squares discriminant analysis (PLS−DA) parameters

Species	A	Accuracy	R^2	Q^2
Rat kidney	5	0.890	0.969	0.802
Rat plasma	3	0.600	0.792	0.500
Human plasma	5	0.909	0.880	0.484

Table S9. The average change of main metabolites (VIP value>1) in kidney contributing for discrimination of control and PB-treated group in PLS-DA models (Mean ± SD)

No.	Metabolites	VIP	3mg/kg		6mg/kg		12mg/kg	
			P value	% Average changes	P value	% Average changes	P value	% Average changes
1	L-Leucine	12.226	$1.12E^{-03}$	85.19±13.79↓	$1.36E^{-03}$	81.47±18.25↓	$4.29E^{-14}$	58.59±7.94↓
2	L-Arginine*	7.4561	$1.77E^{-04}$	76.5±9.74↓	$7.47E^{-03}$	77.75±22.72↓	$2.13E^{-10}$	43.68±10.44↓
3	Malic acid	7.3195	$2.55E^{-01}$	106.89±13.73↑	$5.84E^{-04}$	122.46±12.24↑	$6.23E^{-06}$	141.42±20.65↑
4	L-Proline	6.4736	$2.03E^{-05}$	85.23±9.54↓	$6.34E^{-03}$	87.09±15.6↓	$2.98E^{-11}$	71.61±8.17↓
5	Creatine*	5.0158	$1.02E^{-01}$	82.87±13.19↓	$1.09E^{-02}$	73.72±4.75↓	$1.85E^{-04}$	56.78±9.84↓
6	L-Valine	4.8277	$2.08E^{-01}$	104.22±8.84↑	$1.30E^{-03}$	88.17±8.76↓	$3.61E^{-02}$	91.37±11.87↓
7	Arachidonic acid*	4.5512	$7.02E^{-01}$	89.52±59.12↓	$6.72E^{-01}$	88.95±50.34↓	$2.52E^{-01}$	137.81±88.1↑
8	L-Alanine	4.0796	$2.08E^{-03}$	87.58±10.71↓	$1.09E^{-03}$	84.31±13.57↓	$2.33E^{-11}$	68.13±6.14↓
9	L-Lysine	4.0471	$5.72E^{-03}$	87.28±13.59↓	$3.85E^{-02}$	86.65±21.47↓	$1.58E^{-11}$	63.07±8.59↓
10	Niacinamide	3.8722	$8.17E^{-02}$	94.08±9.45↓	$2.63E^{-02}$	93.36±7.12↓	$8.53E^{-01}$	99.53±5.44↓
11	L-Phenylalanine	3.7419	$3.54E^{-03}$	87.83±12.55↓	$5.97E^{-03}$	85.02±17.48↓	$1.34E^{-11}$	64.51±9.07↓
12	SM(d18:0/24:1) *	3.5724	$2.95E^{-01}$	132.24±60.33↑	$2.87E^{-03}$	185.3±12.91↑	$1.55E^{-04}$	214.97±11.46↑
13	Penicillamine	3.4963	$1.38E^{-03}$	87.89±11.62↓	$4.44E^{-03}$	86.12±15.9↓	$6.77E^{-13}$	68.4±7.16↓
14	Docosahexaenoic acid	3.4849	$3.73E^{-02}$	147.22±69.03↑	$1.53E^{-02}$	164.52±83.07↑	$1.25E^{-02}$	157.81±69↑

15	L-Histidine	3.2803	$7.45E^{-04}$	87.05±11.33↓	$2.12E^{-02}$	87.13±18.82↓	$4.63E^{-10}$	69.66±9.94↓
16	L-Aspartic acid	2.9887	$7.14E^{-04}$	86.72±9.3↓	$8.52E^{-04}$	84.85±12.01↓	$1.26E^{-08}$	72.47±8.49↓
17	Taurine	2.9444	$1.29E^{-02}$	109.91±9.45↑	$3.47E^{-03}$	111.82±9.21↑	$1.03E^{-05}$	123.42±12.18↑
18	LysoPC(16:0) *	2.6192	$7.49E^{-01}$	90.94±47.72↓	$8.94E^{-01}$	96.37±38.22↓	$1.15E^{-01}$	152.62±76.58↑
19	SM(d18:1/24:0) *	2.4738	$6.88E^{-01}$	108.27±44.7↑	$3.26E^{-04}$	171.51±16.71↑	$2.70E^{-05}$	195.72±31.66↑
20	Pipecolic acid*	2.4578	$3.48E^{-03}$	87.88±11.96↓	$5.59E^{-02}$	88.6±20↓	$1.56E^{-11}$	66.44±7.64↓
21	Vigabatrin	2.4525	$4.05E^{-03}$	87.87±12.19↓	$6.42E^{-02}$	88.84±20.24↓	$2.07E^{-11}$	66.34±7.73↓
22	Pantothenic acid	2.3612	$4.49E^{-02}$	90.38±12.69↓	$5.71E^{-01}$	102.59±12.5↑	$1.15E^{-01}$	106.05±7.89↑
23	Octadecanamine	2.3384	$5.28E^{-01}$	103.49±14.47↑	$4.84E^{-01}$	103.92±14.82↑	$2.94E^{-01}$	105.19±10.92↑
24	Hypoxanthine	2.3009	$2.51E^{-01}$	93±17.21↓	$8.99E^{-01}$	99.43±8.63↓	$9.67E^{-01}$	100.2±10.75↑
25	L-Tyrosine	2.11	$8.18E^{-03}$	90.13±11.88↓	$9.62E^{-03}$	87.1±16.5↓	$1.61E^{-12}$	74.23±5.42↓
26	Galactonic acid	2.0651	$8.91E^{-01}$	101.24±29.78↑	$4.16E^{-02}$	87.65±15.34↓	$9.75E^{-01}$	100.22±21.87↑
27	Fumaric acid	2.0039	$1.39E^{-01}$	106.94±12.45↑	$8.28E^{-05}$	120.67±11.62↑	$3.86E^{-06}$	135.15±18.91↑
28	Cytidine	2.0034	$5.52E^{-01}$	96.35±17.67↓	$5.61E^{-01}$	96.33±18.5↓	$2.69E^{-03}$	114.22±7.22↑
29	L-Acetylcarnitine*	1.9816	$5.24E^{-01}$	105.81±23.02↑	$8.49E^{-01}$	102.23±35.86↑	$4.30E^{-02}$	117.03±16.96↑
30	L-Threonine	1.9218	$4.55E^{-03}$	90.63±9.51↓	$1.57E^{-02}$	88.59±15.35↓	$2.22E^{-11}$	72.96±6.31↓
31	Oleic acid*	1.7282	$2.14E^{-01}$	126.19±60.18↑	$1.51E^{-01}$	116.16±41.61↑	$1.67E^{-01}$	123.47±39.02↑
32	LysoPC(18:2) *	1.6926	$2.07E^{-01}$	142.13±86.45↑	$4.27E^{-02}$	164.39±73.02↑	$1.05E^{-02}$	190.28±86.37↑
33	Uracil	1.6417	$2.27E^{-01}$	95.33±10.98↓	$6.51E^{-01}$	101.75±11.23↑	$2.89E^{-02}$	107.11±7.23↑
34	Linoleic acid*	1.6104	$6.14E^{-01}$	115.89±76.09↑	$8.97E^{-01}$	96.28±61↓	$1.83E^{-01}$	143.45±79.56↑
35	L-Glutamine	1.5569	$1.95E^{-01}$	87.8±25.41↓	$2.42E^{-03}$	72.44±19.95↓	$5.37E^{-07}$	43.95±20.85↓
36	Pyruvic acid	1.5418	$5.70E^{-01}$	97.2±15.38↓	$8.67E^{-01}$	100.65±10.7↑	$9.11E^{-01}$	100.49±13.15↑
37	LysoPC(20:4) *	1.5352	$3.65E^{-01}$	118.55±46.71↑	$1.22E^{-01}$	128.68±31.6↑	$6.42E^{-04}$	168.62±28.44↑
38	Stearic acid	1.468	$5.32E^{-02}$	87.41±14.12↓	$2.08E^{-01}$	91.33±17.05↓	$9.78E^{-01}$	99.82±15.65↓
39	Histamine	1.4669	$9.69E^{-01}$	101.15±65.53↑	$7.28E^{-01}$	109.89±60.43↑	$1.53E^{-02}$	181.94±80.33↑
40	Creatinine*	1.409	$3.64E^{-02}$	83.14±18.19↓	$1.20E^{-02}$	139.29±49.58↑	$6.80E^{-05}$	69.6±8.88↓
41	methionine	1.3484	$3.69E^{-03}$	85.73±14.79↓	$5.51E^{-03}$	85.44±16.21↓	$4.04E^{-11}$	64.08±9.1↓
42	L-Asparagine	1.2903	$7.46E^{-02}$	85.16±21.2↓	$1.40E^{-01}$	83.05±35.91↓	$9.07E^{-07}$	46.59±22.23↓
43	L-Serine	1.2825	$1.18E^{-02}$	91.27±10.83↓	$1.36E^{-02}$	89.29±14.17↓	$1.03E^{-09}$	76.26±7.66↓
44	Adenosine	1.2104	$7.13E^{-01}$	105.2±35.97↑	$2.51E^{-01}$	116.13±34.56↑	$4.24E^{-03}$	138.26±24.62↑
45	PC(14:0/22:1) *	1.1536	$1.44E^{-02}$	115.98±15.84↑	$1.26E^{-01}$	109.09±14.05↑	$7.96E^{-02}$	109.11±9.08↑
46	PC(18:0/20:4) *	1.1522	$4.78E^{-01}$	103.26±8.18↑	$3.72E^{-01}$	95.87±8.22↓	$1.09E^{-01}$	92.54±7.82↓
47	L-Carnitine*	1.1504	$9.57E^{-01}$	100.51±27.27↑	$6.01E^{-01}$	104.24±21.31↑	$4.19E^{-01}$	94.75±11.14↓
48	Docosapentaenoic acid	1.1454	$3.56E^{-02}$	136.86±44.24↑	$2.49E^{-02}$	142.99±51.45↑	$1.93E^{-03}$	163.47±52.7↑

49	Lactic acid*	1.1424	2.57E-01	86.3±22.67↓	5.60E-01	106.58±17.21↑	1.72E-01	84.02±19.09↓
50	PC(20:1/14:1) *	1.102	2.11E-01	107.35±17.9↑	5.66E-01	96.71±17.61↓	8.43E-02	92.43±10.42↓
51	3-hydroxy-2-octylpentanedioic acid	1.0951	8.58E-01	98.72±18.8↓	4.53E-01	94.47±19.73↓	9.68E-01	100.3±20.22↑
52	Palmitic acid*	1.0682	8.26E-01	104.17±48.17↑	3.39E-01	82.67±42.49↓	8.67E-01	103.27±51.23↑
53	Ethyl myristate	1.0675	8.24E-01	104.25±48.89↑	3.33E-01	82.35±42.83↓	8.68E-01	103.24±51.22↑
54	LysoPC(18:1) *	1.0554	3.10E-01	123.88±51.03↑	1.67E-01	129.66±35.18↑	4.24E-02	143.48±30.55↑

* shared with rat plasma.

Table S10. The average change of main metabolites (VIP value>1) in rat plasma contributing for discrimination of control and PB-treated group in PLS-DA models (Mean ± SD)

No.	Metabolites	VIP	3mg/kg		6mg/kg		12mg/kg	
			P value	% Average changes	P value	% Average changes	P value	% Average changes
1	LysoPC(18:0)	18.117	4.39E-02	107.38±8.35↑	1.87E-02	108.39±7.54↑	9.02E-05	118.94±10.51↑
2	LysoPC(18:2)*	12.433	2.65E-02	80.24±15.8↓	1.33E-01	85.85±20.44↓	3.60E-03	72.03±15.61↓
3	LysoPC(16:1(9Z))	6.6715	1.08E-02	66.76±24.33↓	2.94E-03	63.59±16.76↓	8.10E-05	45.76±15.7↓
4	PC(18:0/20:4) *	5.6623	4.60E-01	107.49±23.5↑	7.62E-01	102.95±21.42↑	9.17E-02	118.03±23.46↑
5	Oleic acid*	5.3448	6.43E-01	94.21±18.09↓	2.79E-01	86.05±20.09↓	4.81E-02	73.17±18.08↓
6	Creatine*	5.3355	2.05E-01	88.36±23.6↓	4.30E-03	75.32±17.63↓	1.83E-06	51.33±14.91↓
7	LysoPC(17:0)	4.9143	6.27E-03	129.9±24.57↑	4.50E-02	121.46±25.53↑	1.43E-02	132.83±35.28↑
8	Palmitic Acid*	4.4626	5.34E-02	70±34.38↓	4.71E-01	88.21±41.56↓	5.26E-02	68.9±34.74↓
9	SM(d18:0/16:1)	4.4486	4.22E-01	96.6±11.58↓	5.80E-01	102.15±9.99↑	6.65E-04	123.76±19.26↑
10	Arachidonic acid*	4.0239	6.76E-02	129.49±36.85↑	8.34E-01	103.43±41.32↑	4.35E-01	115.38±54.72↑
11	PC(14:1/22:1)	3.3526	6.58E-01	97.97±10.28↓	1.84E-01	106.39±11.08↑	4.44E-02	110.54±11.97↑
12	Lactic acid*	3.3236	5.04E-01	87.89±46.27↓	7.36E-01	106.71±55.13↑	7.28E-01	93.72±44.14↓
13	SM(d18:0/24:1) *	3.154	1.09E-01	111.68±5.29↑	1.01E-01	111.97±5.04↑	1.24E-02	119.72±3.37↑
14	LysoPC(14:0)	2.8426	2.06E-01	77.68±49.6↓	5.91E-03	63.59±22.52↓	1.95E-03	46.39±39.24↓
15	Linoleic acid*	2.6593	6.56E-02	138.77±51.16↑	8.58E-01	103.38±43.73↑	6.74E-01	109.21±56.78↑
16	LysoPE(20:4/0:0)	2.5893	1.51E-02	165.19±72.51↑	2.73E-02	148.94±53.25↑	5.97E-07	244.84±51.69↑
17	Docosapentaenoic acid*	2.5544	1.36E-02	157.75±56.86↑	4.60E-01	113.89±39.97↑	3.13E-01	125.07±67.78↑
18	PC(20:3/20:3)	2.5414	4.89E-01	91.53±37.21↓	2.72E-01	87.46±33.29↓	1.62E-02	121.71±18.67↑
19	PC(14:0/22:1) *	2.4419	5.62E-01	97.96±7.35↓	3.96E-02	107.31±6.56↑	2.80E-04	118.91±11.76↑
20	SM(d18:1/16:0)	2.4407	1.59E-01	94.12±10.38↓	8.84E-01	100.65±12.14↑	2.43E-03	119.52±17.61↑

21	Eicosapentaenoic acid	2.4191	2.63E^{-02}	191.13±119.03↑	2.54E^{-01}	135.56±83.57↑	4.23E^{-02}	169.81±90.53↑
22	L-Carnitine*	2.4018	6.11E^{-01}	96.56±16.29↓	5.70E^{-01}	96.12±16.58↓	3.79E^{-02}	85.5±14.65↓
23	SM(d18:1/24:0) *	2.3479	1.22E^{-01}	112.73±4.83↑	1.11E^{-01}	113.17±5.18↑	1.41E^{-02}	121.88±2.83↑
24	PC(16:1/22:2)	2.2759	3.46E^{-01}	105.77±14.68↑	3.47E^{-01}	105.58±13.62↑	8.41E^{-02}	112.44±18.4↑
25	PC(18:3/16:0)	2.1609	2.16E^{-01}	90.16±19.9↓	3.90E^{-02}	80.96±24.54↓	2.57E^{-03}	75.46±16.12↓
26	LysoPC(20:3)	2.0433	1.37E^{-01}	111.84±18.54↑	9.82E^{-01}	100.19±20.76↑	2.64E^{-01}	109.99±23.02↑
27	L-Acetylcarnitine*	2.0304	8.03E^{-02}	85.45±20.52↓	9.75E^{-01}	99.58±43.51↓	1.78E^{-01}	87.36±25.21↓
28	LysoPC(20:4(8Z,11Z,14Z,17Z)) *	2.0167	6.00E^{-01}	94.73±19.56↓	4.11E^{-01}	92.23±14.8↓	3.59E^{-01}	89.88±23.17↓
29	LysoPE(0:0/18:2)	1.9792	1.43E^{-02}	165.27±75.42↑	2.60E^{-02}	152.24±63.85↑	1.04E^{-04}	193±51.55↑
30	L-Arginine*	1.907	1.03E^{-03}	163.46±34.9↑	1.54E^{-03}	152.72±17.46↑	2.35E^{-02}	148.48±49.41↑
31	LysoPC(O-18:0)	1.8696	7.02E^{-02}	116.26±26.27↑	2.29E^{-04}	134.49±23.45↑	7.41E^{-04}	145.94±38.88↑
32	LysoPC(20:2)	1.8073	8.31E^{-01}	101.92±24.63↑	8.94E^{-01}	98.55±32.97↓	4.22E^{-02}	78.9±28.05↓
33	LysoPC(22:5)	1.7508	1.04E^{-01}	85.08±21.54↓	1.73E^{-01}	84.58±31.92↓	4.26E^{-02}	78.45±26.61↓
34	Palmitoylcarnitine	1.5982	5.35E^{-02}	147.53±71.28↑	1.99E^{-01}	125.43±52.88↑	4.24E^{-03}	178.77±75.92↑
35	Palmitoleic acid	1.5907	6.11E^{-02}	72.18±20.44↓	1.64E^{-01}	78.68±26.04↓	1.98E^{-03}	50.65±13.65↓
36	creatinine*	1.4936	4.91E^{-01}	90.01±34.98↓	2.72E^{-01}	118.23±44.57↑	1.78E^{-04}	197.36±67.5↑
37	12-HETE	1.4714	7.86E^{-03}	320.86±262.12↑	5.35E^{-02}	192.67±143.84↑	6.77E^{-02}	164.75±88.74↑
38	(+/-)11(12)-EET	1.4659	8.04E^{-03}	319.31±261.44↑	5.45E^{-02}	191.65±143.21↑	6.87E^{-02}	176.68±88.56↑
39	Phosphocholine	1.4574	1.08E^{-02}	82.27±15.76↓	5.30E^{-02}	88.39±11.76↓	1.46E^{-03}	76.84±14.82↓
40	Sphinganine	1.4004	1.04E^{-01}	159.7±121.64↑	1.34E^{-01}	132.49±65.14↑	1.41E^{-02}	159.83±70.67↑
41	PC(20:1/14:1) *	1.3495	2.14E^{-02}	87.89±8.23↓	5.16E^{-02}	88.61±12.48↓	4.38E^{-01}	95.34±13.61↓
42	Choline	1.3313	3.98E^{-01}	103.72±13.61↑	4.51E^{-01}	97.61±8.26↓	2.06E^{-02}	90.89±10.48↓
43	11(Z),14(Z)-Eicosadienoic Acid	1.2464	5.60E^{-01}	88.42±43.54↓	3.25E^{-01}	82.09±32.11↓	5.43E^{-02}	63.87±27.02↓
44	PC(O-16:0/18:2)	1.2236	6.00E^{-01}	108.74±40.97↑	3.93E^{-01}	116.27±52.05↑	3.94E^{-03}	156.09±44.19↑
45	11-Eicosenoic acid	1.2064	1.94E^{-01}	86.74±16.83↓	5.52E^{-02}	79.95±16.93↓	1.50E^{-02}	68.82±27.08↓
46	LysoPC(P-16:0)	1.2016	1.93E^{-02}	76.45±21.5↓	5.91E^{-01}	94.52±25.33↓	1.17E^{-01}	83.99±22.66↓
47	(R)-1-Aminopropan-2-ol	1.1917	2.46E^{-01}	109.84±24.67↑	8.41E^{-02}	112.36±18.13↑	2.89E^{-03}	150.51±51.65↑
48	7-Ketolithocholic acid	1.0863	4.13E^{-01}	42.78±13.97↓	3.53E^{-01}	34.99±5.86↓	3.60E^{-01}	32.84±10.08↓
49	Stearoylcarnitine	1.0704	5.27E^{-02}	127.06±33.87↑	2.14E^{-01}	116.33±31.5↑	2.53E^{-03}	157.81±50.18↑
50	Pipecolic acid*	1.0665	6.03E^{-01}	103.1±16.82↑	5.36E^{-02}	108.97±9.26↑	1.45E^{-04}	124.1±13.34↑
51	LysoPC(18:1) *	1.0393	2.37E^{-01}	104.22±9.95↑	7.38E^{-01}	98.76±10.79↓	3.39E^{-02}	93.59±6.39↓
52	LysoPC(16:0) *	1.0307	6.50E^{-01}	98.97±4.94↓	8.06E^{-01}	100.56±5.07↑	3.02E^{-01}	97.22±6.79↓

53	Spermidine	1.0141	4.69E^{-02}	72.45±29.83↓	1.86E^{-03}	57.47±23.55↓	1.07E^{-03}	54.65±20.28↓

* shared with rat kidney.

Table S11. The average change of main metabolites (VIP value>1) in human plasma contributing for discrimination of non-AKI and PB-AKI group in PLS-DA models (Mean ± SD)

No.	Metabolites	VIP	AKI	
			P value	% Average changes
1	LysoPC(18:0)	10.335	2.14E^{-04}	72.44±35.14↓
2	LysoPC(16:0)	9.1951	7.81E^{-03}	83.32±37.63↓
3	PC(16:1/22:5)	8.99	9.81E^{-06}	72.28±28.44↓
4	Creatinine	7.8349	9.08E^{-05}	139.01±52.43↑
5	Hexadecasphinganine	6.5715	1.53E^{-08}	41.38±22↓
6	Caffeine	6.5171	4.34E^{-04}	122.1±37.02↑
7	L-Valine	5.7955	8.83E^{-05}	70.79±43.41↓
8	Creatine	4.9721	1.22E^{-03}	63.12±66.73↓
9	N-Phenylacetylglutamine	4.9282	4.56E^{-04}	273.4±397.93↑
10	Sphinganine	4.2409	1.91E^{-11}	43.19±21.18↓
11	Dopamine	4.0355	3.63E^{-02}	750.83±2666.95↑
12	Decanoylcarnitine	3.826	7.82E^{-03}	173.66±210.75↑
13	Uric acid	3.7904	5.18E^{-04}	134.55±59.81↑
14	L-Octanoylcarnitine	3.2037	1.14E^{-02}	185.36±240.64↑
15	LysoPC(20:4)	3.16	9.76E^{-03}	76.19±51.06↓
16	Butyrylcarnitine	2.9541	6.15E^{-06}	194.47±149.98↑
17	PC(20:3/20:3)	2.86	4.51E^{-05}	70.42±22.17↓
18	Linoleic acid	2.6145	6.07E^{-02}	71.79±61.05↓
19	4-Hydroxy-2-oxo-heptanedioate	2.4758	2.45E^{-06}	179±95.65↑
20	Stearic acid	2.4334	2.34E^{-04}	73.26±30↓
21	PC(14:0/22:5)	2.41	4.75E^{-02}	78.92±44.01↓
22	D-Erythrose	2.3646	1.45E^{-03}	138.92±74.1↑
23	PC(o-16:1/20:4)	2.21	9.63E^{-06}	72.52±31.17↓
24	Hexanoylcarnitine	2.0456	1.68E^{-03}	171.13±176.03↑
25	LysoPE(0:0/18:2)	1.97	1.99E^{-03}	56.12±57.79↓
26	LysoPC(P-16:0)	1.9091	1.30E^{-04}	56.59±46.03↓
27	Geranyl diphosphate	1.8514	2.09E^{-03}	83.72±16.62↓

28	N-Butyryl-L-homoserine lactone	1.7686	3.47E^{-02}	762.52±2741.34↑
29	Propionylcarnitine	1.7309	3.36E^{-02}	130.67±99.3↑
30	PC(o-16:0/22:6)	1.71	2.72E^{-05}	71.74±30.2↓
31	LysoPE(20:4/0:0)	1.68	2.13E^{-04}	59.4±51.89↓
32	D-Xylulose	1.6304	5.81E^{-03}	145.42±83.84↑
33	1-(4-nitrophenyl)piperidine	1.563	1.11E^{-05}	172.3±109.84↑
34	4-(1-adamantyl)-2-methyl-1,3-thiazole	1.5111	1.79E^{-05}	259.16±289.69↑
35	Hydroxypropionylcarnitine	1.4588	3.37E^{-05}	238.27±261.11↑
36	3-Hydroxy-3-methylglutaric acid	1.4209	1.49E^{-02}	120.38±48.15↑
37	3-methylhistidine	1.42	4.04E^{-04}	141.58±60.02↑
38	1-Methylhistidine	1.4149	4.46E^{-04}	141.1±59.38↑
39	LysoPC(17:0)	1.4119	4.78E^{-02}	78.32±63.13↓
40	Aspartylphenylalanine	1.34	1.09E^{-03}	208.99±218.67↑
41	LysoPC(22:5)	1.33	1.61E^{-02}	59.56±64.74↓
42	Testosterone	1.2854	1.18E^{-02}	187.99±248.12↑
43	α-aminoadipic acid	1.24	3.34E^{-06}	177.03±115.59↑
44	N6,N6,N6-Trimethyl-L-lysine	1.22	2.43E^{-03}	136.52±74.88↑
45	pyruvic acid	1.2	4.43E^{-02}	115.29±52.46↑
46	Trimethylamine Hydrochloride	1.2	2.66E^{-02}	287.42±648.98↑
47	1-O-Hexadecyl-lyso-sn-glycero-3-phosphocholine	1.1459	1.25E^{-02}	70.7±56.59↓
48	2,4-Dimethylbenzaldehyde	1.1013	1.23E^{-16}	275.79±151.58↑
49	alpha-Ketoglutaric acid	1.0924	1.07E^{-04}	60.48±35.9↓
50	LysoPC(18:3)	1.09	1.07E^{-02}	72.72±37↓
51	cis-5-Tetradecenoylcarnitine	1.0466	5.74E^{-02}	75.88±64.47↓
52	Oxoadipic Acid	1.0084	3.07E^{-08}	172.04±71.88↑

53	9-HpODE	0.20607	3.11E^{-04}	167.12±131.7↑

Table S12. The average change of main metabolites in rat kidney contributing for discrimination of control and PB-treated group (Mean ± SD)

No.	Compound Name	L (3 mg/kg)		M (6 mg/kg)		H (12 mg/kg)	
		P value	%Average changes	P value	%Average changes	P value	%Average changes
1	cis-Aconitic acid	6.76E^{-01}	106.20±42.67↑	7.23E^{-02}	121.34±28.42↑	1.86E^{-03}	67.76±15.52↓
2	Citric Acid	5.43E^{-01}	97.15±9.91↓	6.61E^{-01}	102.25±12.17↑	3.72E^{-03}	123.26±20.75↑
3	alpha-Ketoglutaric acid	3.07E^{-01}	94.77±15.69↓	6.12E^{-04}	77.05±16.94↓	1.66E^{-07}	59.63±14.35↓
4	Succinic acid	2.88E^{-01}	82.31±13.24↓	1.05E^{-01}	131.77±36.46↑	1.64E^{-01}	72.58±37.94↓
5	Fumaric acid	1.45E^{-01}	106.94±12.45↑	1.03E^{-04}	120.67±11.62↑	2.71E^{-05}	135.15±18.91↑
6	Malic Acid	2.50E^{-01}	106.89±13.73↑	4.80E^{-04}	122.46±12.24↑	1.43E^{-05}	141.42±20.65↑
7	Pyruvic acid	5.87E^{-01}	97.2±15.38↓	8.69E^{-01}	100.65±10.70↑	9.14E^{-01}	100.49±13.15↑
8	Lactic acid	2.43E^{-01}	86.30±22.67↓	5.42E^{-01}	106.58±17.21↑	1.56E^{-01}	84.02±19.09
9	Arginine	1.43E^{-04}	76.50±9.74↓	1.03E^{-02}	77.75±22.72↓	2.30E^{-10}	43.68±10.44↓
10	Proline	1.84E^{-04}	85.23±9.54↓	1.61E^{-02}	87.09±15.60↓	1.09E^{-08}	71.61±8.17↓
11	Histidine	4.91E^{-02}	88.36±16.84↓	2.60E^{-02}	85.22±18.86↓	2.73E^{-09}	67.11±8.74↓
12	D-Glutamic acid	6.42E^{-05}	91.13±4.49↓	9.54E^{-01}	100.20±11.03↑	1.36E^{-03}	88.96±8.86↓
13	L-Glutamine	1.95E^{-01}	87.8±25.41↓	2.42E^{-03}	72.44±19.95↓	5.37E^{-07}	43.95±20.85↓

Table S13. The average change of main metabolites in rat plasma contributing for discrimination of control and PB-treated group (Mean ± SD)

No.	Compound Name	L (3 mg/kg)		M (6 mg/kg)		H (12 mg/kg)	
		P value	%Average changes	P value	%Average changes	P value	%Average changes
1	cis-Aconitic acid	$2.61E^{-01}$	118.14±42.23↑	$4.89E^{-02}$	132.42±41.46↑	$1.32E^{-01}$	135.00±64.9↑
2	Citric Acid	$2.08E^{-01}$	118.12±39.78↑	$1.52E^{-02}$	143.55±48.89↑	$7.94E^{-02}$	177.50±130.17↑
3	alpha-Ketoglutaric acid	$7.29E^{-01}$	105.65±40.38↑	$9.30E^{-02}$	129.05±42.47↑	$4.86E^{-01}$	111.29±37.79↑
4	Succinic acid	$4.23E^{-01}$	83.01±43.24↓	$5.48E^{-01}$	126.58±138.12↑	$6.76E^{-01}$	123.31±172.18↑
5	Fumaric acid	$8.76E^{-01}$	97.25±40.73↓	$8.17E^{-01}$	108.61±118.40↑	$6.87E^{-01}$	116.76±127.59↑
6	Malic Acid	$8.88E^{-01}$	103.12±59.86↑	$3.13E^{-01}$	158.09±185.32↑	$6.06E^{-01}$	136.14±221.06↑
7	Pyruvic acid	$6.61E^{-01}$	94.49±36.67↓	$6.67E^{-01}$	105.41±36.73↑	$9.65E^{-01}$	100.60±39.04↑
8	Histidine	$2.86E^{-01}$	106.83±15.61↑	$1.22E^{-01}$	111.98±20.86↑	$7.82E^{-02}$	110.01±10.73↑
9	Proline	$8.78E^{-01}$	100.97±14.52↑	$9.06E^{-01}$	100.81±17.17↑	$5.12E^{-01}$	96.09±11.98↓
10	Arginine	$9.77E^{-04}$	163.46±34.90↑	$2.03E^{-03}$	152.72±17.46↑	$2.42E^{-02}$	148.48±49.41↑
11	Glutamic acid	$2.39E^{-01}$	130.51±78.98↑	$2.69E^{-01}$	114.54±27.92↑	$8.07E^{-01}$	103.84±39.35↑
