# Supplementary material for: Diagnostic Biomarkers and Metabolic Mechanisms of Polymyxin B-Induced Acute Kidney Injury
Source: Antibiotics (Basel). 2026 Sep 30;15(10):966. doi: 10.3390/antibiotics15100966 (PMC13700662; doi:10.3390/antibiotics15100966)
Supplement: Supplementary file 1 [file antibiotics-15-00966-s001.zip › Supplementary File S2.pdf]

Method validation results of polymyxin B

1. Calibration ranges:

PB1:125-8000ng/mL

ILE-PB1:23.4375-1500ng/mL

PB2:11.71875-750ng/mL

PB3:11.71875-750ng/mL

PB6:7.8125-500ng/mL

LLOQ:

PB1:125ng/mL

ILE-PB1:23.4375ng/mL

PB2:11.71875ng/mL

PB3:11.71875ng/mL

PB6:7.8125ng/mL

2. Internal standards：SPR741 acetate（6000ng/mL)

3. Matrix effects

Table S 2-1. Matrix effects of plasma analytes

compound	QC	matrix effects	RSD（%）
PB1	L	1.76±0.25	14.28
	M	1.26±0.08	6.14
	H	1.14±0.13	11.71
ILE-PB1	L	1.62±0.2	12.35
	M	1.25±0.07	5.65
	H	1.17±0.13	11.07
PB2	L	1.18±0.12	10.59
	M	1.21±0.07	5.52
	H	1.18±0.12	10.13
PB3	L	1.29±0.11	8.68
	M	1.24±0.11	8.57
	H	1.20±0.12	10.01

PB6	L	1.27±0.09	7.31
	M	1.24±0.08	6.06
	H	1.21±0.10	8.07

4. Extraction recovery

Table S 2-2. Extraction recovery of plasma analytes

compound	QC	extraction recovery（%）	RSD（%）
PB1	L	82.23±7.36	8.95
	M	78.84±9.90	12.55
	H	69.61±5.93	8.53
ILE-PB1	L	86.16±7.48	8.68
	M	82.37±9.88	12.00
	H	70.79±5.68	8.02
PB2	L	97.55±10.80	11.07
	M	91.96±8.13	8.84
	H	81.62±6.62	8.11
PB3	L	66.92±8.07	12.06
	M	65.20±6.84	10.49
	H	56.65±3.41	6.03
PB6	L	38.59±5.17	13.39
	M	48.22±3.11	6.44
	H	43.00±3.21	7.45

5. Accuracy and precision

Table S2-3. Accuracy and precision of plasma analytes

compound	QC	Day 1		Day 2		Day 3		Inter-day	
PB1		accuracy(%)	CV(%)	accuracy(%)	CV(%)	accuracy(%)	CV(%)	accuracy(%)	CV(%)
	LLOQ	105.80±9.98	9.43	114.06±3.37	2.95	114.21±3.71	3.25	111.35±6.54	5.87
	L	97.23±5.48	5.64	91.48±5.65	6.18	91.52±5.59	6.11	93.41±5.93	6.35
	M	99.13±2.98	3.01	94.40±6.39	6.77	94.35±6.48	6.87	95.96±5.68	5.92
	H	94.10±5.89	6.26	90.47±3.04	3.36	90.49±3.00	3.32	91.69±4.32	4.71

ILE-PB1	LLOQ	102.79±10.00	9.73	111.48±2.98	2.68	110.31±4.13	3.74	108.19±7.26	6.71
	L	98.27±4.93	5.01	98.98±4.54	4.59	98.97±4.54	4.59	98.74±4.40	4.46
	M	99.84±4.26	4.27	98.27±5.48	5.58	98.26±5.48	5.58	98.79±4.86	4.92
	H	99.41±4.10	4.13	95.89±3.21	3.34	95.88±3.21	3.34	97.06±3.73	3.84
PB2	LLOQ	102.59±21.02	20.49	95.87±9.82	10.25	97.30±9.20	9.46	98.59±13.86	14.06
	L	96.47±6.09	6.31	96.74±3.96	4.09	97.16±3.97	4.08	96.79±4.50	4.64
	M	102.00±2.52	2.47	96.69±3.82	3.95	96.51±4.27	4.42	98.40±4.29	4.36
	H	98.91±3.85	3.89	91.37±1.91	2.10	90.95±1.69	1.86	93.74±4.52	4.82
PB3	LLOQ	88.89±14.26	16.05	109.62±9.81	8.95	109.29±8.24	7.54	102.60±14.41	14.04
	L	98.85±5.88	5.95	99.59±3.44	3.45	98.51±3.36	3.41	98.98±4.15	4.19
	M	103.94±3.03	2.92	98.30±3.78	3.85	97.60±4.93	5.05	99.94±4.75	4.76
	H	99.45±3.45	3.47	93.86±3.28	3.49	92.07±2.74	2.98	95.13±4.40	4.62
PB6	LLOQ	105.88±7.82	7.38	100.44±6.00	5.98	101.08±6.15	6.08	102.47±6.78	6.61
	L	101.08±4.76	4.71	96.03±2.92	3.04	96.05±2.91	3.03	97.72±4.20	4.30
	M	102.12±3.46	3.39	100.16±4.81	4.81	99.84±4.80	4.80	100.71±4.26	4.23
	H	98.67±4.13	4.18	97.29±2.46	2.53	96.95±2.45	2.52	97.64±3.02	3.09

6. Carryover

Injection carryover was assessed by injecting a blank sample following the ULOQ. “No carryover”was confirmed when the peak in the blank at the analyte's retention times was ≤20.0% of the mean analyte response of the LLOQ. Carryover was assessed for PB1-PB6 and found to be less than 20.0% of the LLOQ.
